# Biomimetic Bilayer Hydrogel for Scar-Minimizing Healing of Deep Second–Degree Burns Through Sequential Macrophage–Fibroblast Intervention

**DOI:** 10.3390/gels12100887

**Published:** 2026-10-01

**Authors:** Jian Jia, Jiale Wang, Wenjing Yan, Maosen Yang, Zixuan Zhang, Chaoting Gao, Yang Li, Daidi Fan

**Affiliations:** 1 Engineering Research Center of Western Resource Innovation Medicine Green Manufacturing, Ministry of Education, School of Chemical Engineering, Northwest University, Xi’an 710127, China; 2 Shaanxi Key Laboratory of Biomaterials and Synthetic Biology, Shaanxi R&D Center of Biomaterials and Fermentation Engineering, School of Chemical Engineering, Northwest University, Xi’an 710127, China; 3 Biotech. & Biomed. Research Institute, Northwest University, Xi’an 710127, China

**Keywords:** bilayer hydrogel, second-degree burns, small-interference RNA therapy, scar-minimizing healing

## Abstract

During the inflammatory phase, the persistent macrophage-mediated inflammatory response, followed by excessive fibroblast activation in the proliferative phase, impairs the repair of deep partial-thickness burn wounds and contributes to scar formation. Hence, precise spatiotemporal regulation of both macrophages and fibroblasts is of paramount importance. In this study, we developed a structurally biomimetic bilayer hydrogel system designed to promote scar-minimizing burn wound healing by sequentially targeting macrophages in the inflammatory phase and modulating fibroblast behavior in the proliferative phase. This biomimetic bilayer hydrogel (designated BHM@VP@PPs) consists of a lower layer (LHM) loaded with PBA-PEI@siRNA (TNF-α) nanocomplexes (LHM@PPs) and an upper layer (HHM) encapsulating the small-molecule YAP inhibitor verteporfin (HHM@VP). By varying the HM polymer concentration and crosslinking density, the bilayer hydrogel was designed to provide degradation-dependent, stage-matched release of siRNA and VP. During the inflammatory phase, released siRNA silenced TNF-α via blockade of its mRNA expression, thereby attenuating local inflammation and accelerating the transition to the proliferative phase. Upon degradation of the lower layer, the upper layer subsequently released VP, which suppressed excessive fibroblast activation through YAP-related signaling pathways during the proliferative phase, thus favoring scar-minimizing healing. Moreover, VP-mediated photodynamic therapy (PDT) generated abundant reactive oxygen species (ROS), effectively eradicating bacteria and preventing infection. Collectively, this bilayer hydrogel system synergistically facilitates scar-minimizing repair of deep second-degree burn wounds, offering a promising therapeutic strategy for clinical translation.

## 1. Introduction

Globally, burns are the third most common type of trauma, after road traffic accidents and physical violence [1]. They are also among the most clinically fatal, disabling, and disfiguring traumatic conditions, with a high propensity for severe infection [2]. According to World Health Organization estimates, 11 million people require medical treatment for burns annually, and 180,000 deaths result from burns worldwide. Deep second-degree burns involve the epidermis and the deep layers of the dermis, and their healing process is often accompanied by impaired epithelial regeneration, scar formation, and functional deficits [3]. Therefore, the management of deep second-degree burn wounds goes beyond simple wound coverage; it aims to achieve high-quality healing while minimizing post-healing scarring, functional impairment, and cosmetic disfigurement [4,5,6].

Wound repair is a highly coordinated and programmed biological process. The general healing process typically progresses through three distinct yet overlapping phases [7,8]: the inflammatory phase, the proliferative phase, and the remodeling phase. Disruption or dysfunction in any of these phases can adversely affect healing. During the inflammatory phase, macrophages undergo aberrant polarization and release large quantities of cytokines, such as tumor necrosis factor-α (TNF-α) and interleukins [9]. This leads to an imbalance in the regulation of inflammatory factors at the wound site, resulting in a prolonged inflammatory state and creating a persistently dysregulated immune microenvironment. Consequently, wound repair fails to transition normally into the proliferative phase, thereby impeding the healing process [10]. Studies have shown that deep second-degree burn wounds that remain in a prolonged inflammatory phase may induce excessive activation of proliferating fibroblasts into myofibroblasts, leading to excessive deposition and disorganized arrangement of collagen in the extracellular matrix and ultimately resulting in pathological scarring [7,9,11,12,13,14]. Current strategies to inhibit scar formation include regulating macrophages to suppress inflammation or intervening in fibroblasts to block fibrotic pathways [15]. However, these approaches do not align with the temporal sequence of wound healing to achieve coordinated regulation of both cell types. Therefore, based on the natural temporal pattern of wound repair, there is an urgent need to develop a regulatory strategy that specifically targets macrophages during the inflammatory phase and fibroblasts during the proliferative phase, thereby promoting scar-minimizing healing of deep second-degree burn wounds.

Gene therapy [16], particularly small interfering RNA (siRNA) technology based on RNA interference (RNAi), achieves post-transcriptional gene silencing by specifically degrading target messenger RNA (mRNA) [17,18]. This approach has demonstrated potential for precision therapy in regulating genes associated with inflammatory factors. To date, more than ten siRNA drugs have been approved by the U.S. Food and Drug Administration (FDA) for the treatment of genetic disorders and chronic diseases [19,20]. However, naked siRNA exhibits poor stability in physiological environments, is susceptible to nuclease degradation, lacks active targeting capability, and struggles to cross cell membranes and endosomal barriers [21,22,23]. Therefore, there is an urgent need to develop safe and efficient delivery vehicles. Non-viral delivery systems, such as the cationic polymer polyethyleneimine (PEI), have been extensively studied due to their low immunogenicity and the “proton sponge effect” [24,25,26,27,28]. Among these, branched PEI with a molecular weight of 25 kDa is regarded as the benchmark for transfection efficiency [29]. However, high transfection efficiency is often accompanied by high cytotoxicity, and PEI lacks the ability to recognize specific cell types. Phenylboronic acid (PBA) and its derivatives carry a negative charge and can specifically target sialic acid residues on the surface of macrophages. Grafting PBA onto PEI not only reduces the toxicity of PEI but also has the potential to endow it with active macrophage-targeting capability, providing a new strategy for siRNA delivery [30].

On the other hand, the Hippo signaling pathway plays a key role in regulating tissue growth and fibrosis [31,32,33]. Its downstream effectors, YAP/TAZ, are closely associated with pro-fibrotic signaling pathways and can drive the transformation of fibroblasts into myofibroblasts, leading to abnormal collagen deposition and scar formation [34,35,36]. Verteporfin (VP) is a photosensitizer approved by the FDA for the treatment of age-related macular degeneration. Recent studies have shown that VP can precisely inhibit YAP activity, achieving scar-minimizing healing in animal models [37,38,39,40,41,42]. Furthermore, photodynamic therapy (PDT) mediated by VP under 660 nm red light irradiation efficiently generates abundant reactive oxygen species (ROS), thereby endowing the system with prominent antibacterial performance [43,44,45,46]. Therefore, the combined application of VP and siRNA is expected to synergistically promote the repair of deep second-degree burn wounds through both anti-inflammatory and anti-fibrotic mechanisms. A key question remains: how can the stage-matched delivery of these two agents be achieved to enable temporally synergistic regulation and promote scar-minimizing healing of burn wounds?

In this study, a skin-mimetic methacrylate hyaluronic acid (HM) bilayer hydrogel was designed to promote scar-minimizing healing of burn wounds. By adjusting the concentrations of the upper and lower layers and the photopolymerization time, a bilayer structure with distinct crosslinking degrees was obtained. During the inflammatory stage, PBA-PEI@siRNA nanocomposites loaded in the lower hydrogel layer (LHM) are released and act on infiltrated macrophages. This intervention suppresses the mRNA expression of TNF-α, alleviates excessive inflammatory responses, and facilitates the transition of wound healing from the inflammatory phase to the proliferative phase. Upon degradation of the lightly crosslinked lower layer, VP released from the upper layer intervenes in the YAP-related signaling pathway, inhibiting the differentiation of fibroblasts into myofibroblasts. Furthermore, under photodynamic therapy (PDT), VP generates reactive oxygen species (ROS), conferring antibacterial activity. Collectively, this bilayer hydrogel system enables sequential modulation of macrophage inflammatory signaling during the inflammatory phase and fibroblast fibrotic pathways during the proliferative phase, ultimately promoting scar-minimizing healing of deep second-degree burn wounds. This study provides a novel therapeutic strategy for the clinical management of deep second-degree burns (Scheme 1).

## 2. Results and Discussion

### 2.1. Synthesis and Characterization of PBA-PEI@siRNA

The preparation process of the PBA-PEI@siRNA nanocomposites is illustrated in Figure 1A. The cytotoxicity of PBA-PEI polymers at different concentrations against RAW 264.7 cells was evaluated using the CCK-8 assay. By introducing negatively charged PBA groups, the positive charge density of the polyethyleneimine (PEI) amino groups was partially neutralized. Compared with unmodified PEI, PBA-modified PEI retains the siRNA delivery capability of PEI while maintaining a cell viability of 70% at a concentration of 6 µg/mL (Figure 1B). These results indicate that PBA-PEI exhibits good biocompatibility while retaining the functional properties of PEI. Furthermore, the cytotoxicity of HM hydrogel extracts at different concentrations against RAW 264.7 cells was also assessed using the CCK-8 assay (Figure 1C). Neither the lower-layer nor the upper-layer hydrogels showed significant cytotoxicity toward the tested cells, suggesting that the HM hydrogel is a safe and mild drug carrier. A hemolysis assay was conducted to evaluate blood compatibility. The hemolysis rate in the positive control group (dd H_2_O) was 100%, whereas the hemolysis rates in all experimental groups were well below 5% (Figure 1D), demonstrating that the bilayer hydrogel possesses good hemocompatibility and biosafety.

Comparison of the FT-IR spectrum of PEI and PBA-PEI (Figure 1F) revealed that PBA-PEI exhibited an absorption peak at 1408 cm^−1^ corresponding to the boronic acid group of phenylboronic acid, a peak at 1507 cm^−1^ corresponding to the C=C vibration of the phenyl ring, a peak at 1174 cm^−1^ for the benzene ring stretching vibration, and a peak at 1608 cm^−1^ for the amide group of polyethyleneimine. The ^1^H NMR spectrum (Figure 1E) showed that PBA-PEI displayed proton peaks of PEI at δ = 2.34–2.85 ppm, as well as proton peaks of the benzene ring at δ = 7.69, 7.39, 3.76, and 3.66 ppm. These results collectively confirm the successful preparation of PBA-PEI. Elemental analysis of the PBA-PEI polymer was performed for C, H, and N (Table S1). The theoretical nitrogen content of PEI is 32.5%, while the measured nitrogen content of PBA-PEI was 21.55%. The grafting ratio of PBA-PEI was calculated to be approximately 50.8%.

It is generally recognized that positively charged nanoparticles with particle sizes in the range of 150–300 nm are more readily taken up by macrophages. As shown in Figure 1H, as the N/P ratio (M = nitrogen/phosphate mass ratios) increased from 1 to 7.5, the particle size of the PBA-PEI@siRNA nanocomposites gradually increased from 175 nm to 230 nm, with most particles clustering around 200 nm, which falls within the optimal size range for cellular uptake. Because siRNA carries a negative charge and tends to be electrostatically repelled by the cell membrane, it cannot efficiently enter cells on its own. However, PBA-PEI effectively encapsulated the siRNA, converting the surface charge from negative to positive (Figure 1I). As the N/P ratio increased from 1 to 10, the zeta potential of the nanocomposites gradually rose from −2.2 mV to 17.5 mV. Considering both particle size and zeta potential, an N/P ratio of 7.5 was selected as the optimal value, which is expected to result in high cellular transfection efficiency. The microstructure of the nanocomposites was observed using TEM (Figure 1G). The results showed that the PBA-PEI@siRNA nanocomposites were spherical, with a particle size of approximately 230 nm, which is consistent with the particle size measured by DLS.

Due to its negative charge, free siRNA exhibited a clear, bright band in agarose gel electrophoresis. In contrast, the control group containing only PBA-PEI showed no band (Figure 1J). PBA-PEI binds to siRNA via electrostatic interactions, effectively blocking its migration in the electric field. As the N/P ratio increased, the intensity of the siRNA band gradually decreased; when the N/P ratio exceeded 5, the band disappeared completely, indicating that the siRNA was fully retained within the gel pores. These results demonstrate that PBA-PEI possesses the fundamental properties required for an siRNA delivery vehicle. Therefore, all subsequent cell transfection experiments were conducted at an N/P ratio of 7.5. After incubation for approximately 3 h in media containing 10% or 50% fetal bovine serum, the electrophoresis bands of free siRNA almost completely disappeared, indicating that it had been fully degraded. In contrast, after 24 h of incubation under the same serum conditions, distinct siRNA bands were still detectable for the PBA-PEI@siRNA nanocomposites (Figure S1), suggesting that the cationic PBA-PEI polymer effectively protects siRNA from nuclease degradation prior to cellular entry. Quantitative analysis of the band intensities using ImageJ software (Figure S2) showed that after 24 h of incubation in 10% and 50% serum, 76.65% and 64.50% of the siRNA band intensity, respectively, were retained.

### 2.2. Characterization of HM

The schematic preparation process of the HM bilayer hydrogel is illustrated in Figure 2A. The FT-IR spectra of both HA and HM showed characteristic absorption peaks at approximately 3300, 2900, 1600, and 1400 cm^−1^, corresponding to O–H/N–H stretching vibrations, C–H stretching vibrations, C=O asymmetric stretching vibrations, and C–H bending vibrations, respectively. Additionally, both materials exhibited a strong absorption band at approximately 1040 cm^−1^, attributed to C–O–C bending vibrations. Compared with HA, the FT-IR spectrum of HM displayed new absorption peaks at 1740 and 850 cm^−1^, corresponding to the C=O stretching vibration and the C=C stretching vibration of the conjugated system, respectively. These new peaks confirm the successful grafting of the double bond from methacrylic anhydride (MA) onto the HA molecular chain (Figure S3). In the ^1^H NMR spectrum of native HA, the chemical shift in the HA methyl proton appeared at 1.9 ppm. After MA modification, the ^1^H NMR spectrum of HM exhibited characteristic peaks of the methacrylate vinyl protons at 5.6 and 6.1 ppm, while the peak at 1.8–1.88 ppm was primarily attributed to the methyl protons adjacent to the double bond. These results confirm the successful synthesis of HM (Figure 2B).

### 2.3. Characterization of the Hydrogels

SEM images revealed that the BHM@VP@PPs hydrogel possessed a porous and interconnected network structure, with significant differences in pore size between the upper and lower layers. The lower layer exhibited larger pores, which facilitated the absorption of tissue exudate; the upper layer displayed smaller pores and a denser network structure, which not only confers excellent water-retention capacity to the hydrogel but also effectively blocked external bacteria, serving as an anti-adhesion physical barrier (Figure 2C). Swelling rate tests showed that the swelling rates of LHM, BHM, and HHM hydrogels after 24 h were 360%, 280%, and 240%, respectively (Figure 2D), indicating that the bilayer hydrogel can effectively absorb wound exudate. In vitro degradation experiments demonstrated that LHM degraded almost completely within approximately 60–75 h, while HHM retained approximately 35–40% of its initial mass after 250 h (Figure 2E). As the concentration of the HM solution increased, the compressive mechanical properties of the LHM, BHM, and HHM hydrogels gradually increased (Figure 2F). By optimizing the concentration ratio between the upper and lower layers, the bilayer hydrogel avoided the molding difficulties and structural instability associated with low concentrations, while also overcoming the increased brittleness and fragility caused by high concentrations. Thus, the hydrogel exhibited good structural integrity and functional adaptability for wound dressing applications.

Rheological measurements were performed to evaluate the viscoelastic properties of the hydrogels. Under a constant angular frequency of 10 rad/s with gradually increasing shear stress, higher-concentration hydrogels exhibited higher storage modulus ($G^{\prime }$) and loss modulus ($G^{\prime \prime }$). As shear stress increased, the HHM hydrogel reached the intersection of $G^{\prime }$ and $G^{\prime \prime }$ first, followed by BHM, with LHM reaching the intersection last (Figure 2G). In rheology, when $G^{\prime }\geq G^{\prime \prime }$, the material is in a stable gel state; when $G^{\prime }<G^{\prime \prime }$, it transitions to a fluid state, and the network structure breaks down. The intersection strain of the low-concentration LHM hydrogel (385%) was significantly higher than that of HHM (235%). The intersection strain of BHM lay between the two, reflecting moderate mechanical strength and flexibility. Angular frequency sweeps (0.1–100 rad/s) were conducted under a constant shear strain (1%). The results showed that high-concentration hydrogels still exhibited higher $G^{\prime }$ and $G^{\prime \prime }$. In contrast to the strain sweep results, during the frequency sweep, the $G^{\prime }$ and $G^{\prime \prime }$ of all hydrogels remained essentially constant with increasing angular frequency, and no intersection between $G^{\prime }$ and $G^{\prime \prime }$ was observed (Figure 2H). This indicates that under constant shear strain and varying angular frequency, the hydrogels consistently maintained a stable gel state. In the absence of UV irradiation, the $G^{\prime \prime }$ of the hydrogel precursor was significantly higher than the $G^{\prime }$, indicating a fluid state in which an effective cross-linked network had not yet formed between the molecular chains.

The point at which $G^{\prime }\geq G^{\prime \prime }$ is defined as the gelation critical point. After 12 s of UV irradiation, $G^{\prime }$ rapidly increased and exceeded $G^{\prime \prime }$ (Figure 2I). LHM, BHM, and HHM all exhibited rapid gelation behavior in the early stage of irradiation, indicating that the cross-linking reaction proceeded rapidly under UV irradiation, enabling the fast formation of a stable gel structure. Further comparison of the effects of different irradiation conditions on the mechanical properties revealed that, compared with samples irradiated for only 12 s (Figure S4), the storage modulus of all hydrogels significantly increased under prolonged UV irradiation. This suggests that extending the irradiation time promotes a more complete cross-linking reaction, leading to a denser network structure and consequently a higher elastic modulus.

Based on these properties, the BHM@VP@PPs hydrogel can rapidly degrade the lower layer (LHM@PPs) during the early inflammatory phase and release the PBA-PEI@siRNA nanocomposite, thereby alleviating the local inflammatory response at the burn wound site. In the late healing phase, the upper hydrogel (HHM@VP) slowly releases the YAP inhibitor VP, inhibiting the excessive differentiation of fibroblasts into myofibroblasts and thus reducing scar formation. In summary, the BHM@VP@PPs hydrogel developed in this study enables sequential spatiotemporal drug release and promotes the healing of deep second-degree burn wounds through the synergistic effects of anti-inflammatory and anti-scarring mechanisms.

### 2.4. Gene Silencing Efficiency of PBA-PEI@siRNA Nanocomposites

#### 2.4.1. Cellular Uptake of PBA-PEI@siRNA Nanocomposites

FAM-labeled siRNA (green fluorescence) was used to track the cellular uptake of the nanocomposites. RAW 264.7 cells were incubated with PBA-PEI@siRNA (FAM-labeled) nanocomposites for a defined period, and endocytosis was observed under an inverted fluorescence microscope. The phagocytic uptake efficiency of RAW 264.7 macrophages for nanocomposites at different N/P ratios was evaluated. Free siRNA alone was poorly internalized by the cells. In contrast, upon complexation with the cationic polymer PBA-PEI, the nanocomposites effectively traversed the cell membrane, and the internalization efficiency increased with increasing N/P ratio (Figure S5). However, at an N/P ratio of 10, extensive cell death was observed, which was attributed to the presence of excess unbound PBA-PEI cationic polymer exerting significant cytotoxicity.

Semiquantitative analysis of the fluorescence images was performed using ImageJ software (Figure S6). The free siRNA group exhibited only weak fluorescence, indicating poor uptake by macrophages. The uptake of PBA-PEI@siRNA (FAM-labeled) nanocomposites increased with the N/P ratio, reaching a maximum at an N/P ratio of 7.5. At this ratio, the nanocomposites had a particle size of approximately 230 nm and carried a positive charge, demonstrating that the PBA-PEI cationic polymer effectively delivered siRNA into macrophages.

To further quantify the uptake efficiency, RAW 264.7 cells were treated with FAM-labeled free siRNA or PBA-PEI@siRNA nanocomposites at various N/P ratios for 12 h, and the intracellular fluorescence was analyzed by flow cytometry. The free siRNA control group showed weak fluorescence, whereas the PBA-PEI@siRNA-treated groups exhibited significantly enhanced fluorescence (Figure 3A). Semiquantitative analysis (Figure 3B) revealed that the fluorescence intensity gradually increased with rising N/P ratios, reaching saturation at an N/P ratio of 7.5. Further increasing the N/P ratio to 10 reduced the uptake efficiency, likely due to increased cytotoxicity leading to cell death.

#### 2.4.2. Gene Silencing Efficiency of TNF-α

To quantitatively evaluate the gene silencing efficiency of the PBA-PEI@siRNA nanocomposite targeting TNF-α, LPS-stimulated RAW 264.7 cells were used as a model. Transfections were performed with nanocomposites at different N/P ratios, and TNF-α expression was assessed at both the mRNA and protein levels using RT-qPCR and ELISA, respectively. Negative control siRNA (NC), GAPDH, and blank control groups were included. LPS stimulation significantly upregulated TNF-α expression, whereas delivery of TNF-α targeted siRNA effectively suppressed its expression (Figure 3C). At an N/P ratio of 7.5, the PBA-PEI@siRNA group showed a significant reduction in TNF-α mRNA expression compared with the NC and GAPDH groups, confirming specific gene silencing by the designed siRNA sequence. Comparison of silencing efficiency across different N/P ratios (Figure 3D) showed that the inhibitory effect increased with rising N/P ratios, peaking at N/P = 7.5. RT-qPCR and ELISA results were consistent: at N/P = 7.5, TNF-α expression was reduced by approximately 70% at the mRNA level and by approximately 55% at the protein level.

#### 2.4.3. Anti-Inflammatory Effect of Hydrogels

The protein levels of inflammatory factors in the supernatant of LPS-stimulated RAW 264.7 cells were measured by ELISA to evaluate the anti-inflammatory effect of the PBA-PEI@siRNA nanocomposites. The LPS-stimulated group served as the positive control (inflammatory model), and the untreated group as the blank control. Compared with the blank control, the LPS group showed significantly elevated expression of the pro-inflammatory cytokines TNF-α and IL-6, supporting the successful establishment of the inflammatory model. Treatment with the LHM@PPs hydrogel significantly reduced the protein levels of TNF-α and IL-6 and increased the expression of the anti-inflammatory cytokines IL-10 and TGF-β relative to the LPS group (Figure 3E–H). These results suggest that LHM@PPs hydrogel may help regulate the expression of inflammatory factors in macrophages and may possess considerable anti-inflammatory potential, potentially alleviating local inflammation at the wound site and supporting early wound healing. Furthermore, under LPS stimulation, the fluorescence intensity of the M2 marker CD206 in macrophages increased only slightly. However, after treatment with the LHM@PPs hydrogel, the CD206 positivity rate in RAW 264.7 cells significantly increased to 14.6% (Figure 3I), and the levels of anti-inflammatory factors were approximately threefold higher than those in the LPS group. To further assess macrophage polarization and the shift away from classical pro-inflammatory macrophage state towards an anti-inflammatory, pro-repair functional profile, immunofluorescence staining of specific biomarkers was performed on RAW 264.7 cells (Figure 3J). The LPS group exhibited the strongest fluorescence intensity for the M1 marker CD86. In contrast, after treatment with the LHM@PPs hydrogel, RAW 264.7 cells displayed strong CD206 fluorescence. Collectively, these results suggest that the PBA-PEI@siRNA nanocomposites loaded into the LHM@PPs hydrogel could deliver TNF-α siRNA into the cytoplasm to induce gene silencing, and thereby potentially alleviate excessive local inflammatory responses.

### 2.5. Release Behavior of Hydrogels

Using a 96-well plate and multi-mode microplate reader, we measured the fluorescence intensity of siRNA at 490 nm and the characteristic absorbance of VP at 689 nm and plotted the release profiles of siRNA and VP (Figure S7). The results revealed that siRNA loaded in the lower hydrogel layer exhibited rapid release at early time points, with substantial release within 12 h and near-complete release equilibrium achieved at approximately 48 h. This release profile closely matches the required timeline for timely anti-inflammatory intervention during the early stage of wound healing. In contrast, VP encapsulated in the upper layer displayed a delayed release pattern: its release remained extremely low within the first 48 h with an obvious lag phase, followed by a steady-state release lasting around 7 days. Collectively, this bilayer hydrogel system achieves rapid drug release in the early stage and subsequent slow, sustained delivery, enabling the sequential, ordered release of siRNA and VP. Such design avoids disordered drug release caused by mutual interference between the two agents.

### 2.6. In Vitro ROS Generation by VP

#### 2.6.1. DCFH-DA Fluorescence Assay

The ability of VP to generate ROS under 660 nm red light irradiation was evaluated using DCFH-DA as a fluorescent probe (Figure S8). In a mixture containing DCFH-DA and VP, the amount of ROS generated gradually increased with prolonged irradiation time. In contrast, the control solution containing DCFH-DA alone produced only trace amounts of ROS under the same irradiation conditions. These results indicate that ROS generation primarily originates from VP-mediated photochemical reactions during PDT. Notably, the absorption spectra of the VP-containing mixture in the 300–900 nm range were measured before and after red light irradiation (Figure S9). The characteristic absorption peaks showed only slight changes, suggesting that VP may act as a catalyst during the PDT process, with its chemical structure remaining essentially stable before and after the reaction.

#### 2.6.2. Electron Paramagnetic Resonance (EPR) Spectroscopy

EPR spectroscopy was employed to characterize the types of ROS generated by VP under 660 nm red light irradiation, with a focus on detecting changes in the signals of singlet oxygen (^1^O_2_) and superoxide anion (·O_2_^−^) (Figure 4A and Figure S10). Under dark conditions, the signal intensity of ^1^O_2_ was 9; after 10 min of PDT irradiation, it increased to 30, approximately three-fold higher. The signal intensity of ·O_2_^−^ was 0.005 under dark conditions and increased to 0.25 after irradiation, approximately 50-fold higher. Although the relative increase in the ·O_2_^−^ signal was greater, its absolute intensity remained far lower than that of ^1^O_2_. Thus, the ROS generated by VP under PDT are primarily ^1^O_2_, with ·O_2_^−^ as a secondary component. Moreover, PDT-induced ROS production, mainly consisting of ^1^O_2_, effectively relieves delayed wound healing resulting from bacterial infection.

#### 2.6.3. DPBF Chemical Probe Assay

DPBF was used as a chemical probe to assess ^1^O_2_ generation. The changes in the absorption spectra of the mixed solution in the 300–500 nm range were examined under different irradiation times and irradiance levels. Under a fixed irradiation power of 25 mW/cm^2^ (Figure 4B), the intensity of the absorption peak at 420 nm gradually decreased with increasing red light irradiation time, indicating increased ^1^O_2_ production. The absorption peak was highest in the non-irradiated sample and dropped to its lowest after 15 min of irradiation. Under a fixed irradiation time of 15 min (Figure 4C), as the light intensity increased from 0 to 50 mW/cm^2^, the intensity of the 420 nm absorption peak also gradually decreased, indicating that the amount of ^1^O_2_ generated is positively correlated with light intensity. To verify the consistency of the VP content in the mixed solution under different testing conditions, the full-wavelength absorption spectra (300–800 nm) of the mixed solution were measured (Figures S11 and S12). Under conditions of fixed light intensity and varying irradiation times, the peak intensity at 689 nm remained essentially consistent. Similarly, under conditions of fixed irradiation time and varying light intensity, the 689 nm peak intensity showed no significant change. These findings indicate that the chemical properties of VP remain unaltered in mixed solutions under the tested experimental conditions. Even upon 660 nm light irradiation, VP still efficiently inhibits the transformation of fibroblasts into myofibroblasts.

#### 2.6.4. Characterization of the ROS-Generating Properties of Hydrogels

The ability of the HHM@VP hydrogel to generate ROS under PDT conditions was evaluated using the DCFH-DA probe. Fluorescence staining of ROS in mouse embryonic fibroblasts (3T3) (Figure 4D,E) showed that the blank control group and the HHM group without VP loading exhibited only weak fluorescence. The VP + L group showed a strong fluorescence signal. The HHM@VP + L group also exhibited strong fluorescence intensity, though slightly lower than that of the VP + L group, which indicated that released VP was still capable of generating a large amount of ROS under PDT irradiation. Compared with the control group, the fluorescence intensity difference in the HHM@VP + L group was statistically significant. These results indicate that VP can effectively generate substantial ROS under 660 nm (25 mW/cm^2^) red light. To further validate the in vitro ROS-generating capacity of VP, flow cytometry was performed on the different treatment groups (Figure 4F,J). The blank control group and the HHM-only group exhibited only very weak fluorescence signals under 660 nm red light (25 mW/cm^2^), whereas both the VP + L group and the HHM@VP + L group demonstrated significantly enhanced fluorescence intensity. These results are consistent with observations obtained under an inverted fluorescence microscope, confirming that VP can generate abundant ROS upon PDT irradiation.

### 2.7. In Vitro Antimicrobial Activity of Hydrogels

The antimicrobial efficacy of different treatments against *Staphylococcus aureus* (*S. aureus*) was evaluated using the plate colony counting method. Bacterial suspensions from each treatment group were evenly spread onto solid agar plates and incubated under identical conditions (37 °C for 24 h). The number of colony-forming units (CFUs) was then determined (Figure 4G). Compared with the control group, the PDT-only group (L) showed no significant reduction in colony count, indicating that photodynamic therapy alone lacks antibacterial activity. Similarly, the HHM hydrogel-only group under PDT treatment (HHM + L) also exhibited no significant antibacterial effect, suggesting that the hydrogel itself, without VP loading, has no bactericidal activity. In contrast, the VP + L group and the HHM@VP + L group showed a marked reduction in colony counts compared with the control group, with statistically significant differences. These results indicate that VP-containing samples exhibit potent antibacterial activity after PDT treatment.

To further validate the antibacterial efficacy and observe structural damage to bacteria, SEM was performed to examine the morphology of bacteria in different treatment groups (Figure 4G and Figure S13A). In the control group, *S. aureus* and *E. coli* displayed a smooth surface and intact cell wall. Bacteria in the PDT-only group exhibited a similar morphology, indicating that light irradiation alone did not disrupt bacterial structure. In the HHM-only group, the bacterial surface appeared slightly rough, but the overall structure remained intact, suggesting that the hydrogel itself caused only limited damage. In contrast, bacteria in the VP + L and HHM@VP + L groups underwent significant morphological changes: some bacterial surfaces showed wrinkles, indentations, and cracks, and even cell collapse and cell wall rupture were observed, indicating severe structural damage. These findings confirm that VP, under PDT, possesses a potent bactericidal effect against *S. aureus* and *E. coli*.

Quantitative analysis of bacterial survival was further performed by measuring the optical density (OD) of the bacterial suspensions (Figure 4H,I and Figure S13B,C). The OD values and bacterial survival rates in the VP + L and HHM@VP + L groups were significantly lower than those in the control group and other treatment groups, with statistically significant differences. These results are highly consistent with the plate counting and SEM observations, further confirming that VP generates substantial ROS through the photodynamic effect, effectively destroying bacterial cell structures. In summary, the HHM@VP hydrogel combined with PDT exhibits excellent in vitro antibacterial performance, significantly inhibiting *S. aureus* and *E. coli* growth, and provides an effective physicochemical synergistic strategy for controlling burn wound infections.

It has been reported that the small-molecule inhibitor verteporfin (VP) suppresses YAP protein expression by interfering with the YAP/TAZ signaling pathway and reduces the activation level of the Engrailed-1 gene [47,48,49,50,51]. Inhibition of the YAP/TAZ pathway further decreases the production of α-smooth muscle actin (α-SMA) in fibroblasts. α-SMA is a key marker of fibroblast-to-myofibroblast activation, and its high expression is closely associated with wound scarring. The cytotoxicity of verteporfin is presented in Figure S14. Then to evaluate the anti-scarring effect of VP, Western blotting was performed to detect YAP and α-SMA protein expression in fibroblast cells treated with different concentrations of VP (0, 1, 2, and 5 μg/mL), with untreated cells serving as the blank control (Figure S15). Grayscale analysis of the protein bands was conducted using ImageJ software (Figure S16). The results showed that as the VP concentration increased, the expression levels of YAP and α-SMA proteins gradually decreased. Compared with the control group (0 μg/mL), the VP-treated groups exhibited a downregulated trend in protein expression, and the difference reached statistical significance in the 5 μg/mL VP-treated group.

### 2.8. In Vivo Animal Models for Hydrogels

#### 2.8.1. Wound Healing

To evaluate the therapeutic efficacy of the BHM@VP@PPs hydrogel combined with photodynamic therapy (PDT) for deep second-degree burn wounds, a murine deep second-degree burn model was constructed and subjected to corresponding treatments (Figure 5A). The experimental groups included saline control, LHM@PPs, HHM@VP, HHM@VP + L, BHM@VP@PPs, and BHM@VP@PPs + L. Wound healing progression was monitored at scheduled time intervals (Figure 5B), and wound areas were quantitatively analyzed via image analysis software (Figure 5C,D). Continuous observation of wound healing trajectories clearly demonstrated that the BHM@VP@PPs + L group achieved the most rapid healing outcome. Quantitative statistical analysis further confirmed that on day 15 post-treatment, the wound contraction rate of the BHM@VP@PPs + L group reached approximately 85%, which was significantly higher than those of the control group (60%), LHM@PPs group (70%), HHM@VP group (65%), HHM@VP+L group (75%), and BHM@VP@PPs group (80%). By day 21, wounds in the BHM@VP@PPs + L group achieved nearly complete closure. This superior healing performance can be attributed to the unique synergistic regulation of cellular behaviors during the inflammatory and proliferative phases by the bilayer hydrogel system, which efficiently accelerates the healing process of burn wounds.

#### 2.8.2. Histological Analysis

H&E staining (Figure 6A) showed that on day 7 post-treatment, the epidermal structure was markedly disrupted in all groups, confirming successful establishment of the deep second-degree burn model. On day 15, the epidermal tissue remained damaged in the other groups, whereas the BHM@VP@PPs + L group was the first to exhibit newly formed epidermal tissue. On day 21, skin appendages in the other groups had not yet fully formed; in contrast, the wound in the BHM@VP@PPs + L group had completely healed, with abundant new blood vessels and hair follicle structures, and the regenerated skin displayed a more natural and mature morphology.

Masson’s trichrome staining revealed that by day 15, the wound in the BHM@VP@PPs + L group had completely healed with partial collagen fiber deposition (Figure 6B). By day 21, abundant collagen fibers and rich skin appendages (hair follicles) were observed. In the other groups, only minimal collagen depositions and no obvious skin appendages were seen.

#### 2.8.3. Early Angiogenesis

To assess early wound angiogenesis, VEGF immunofluorescence staining (red) was performed on wound tissues collected on day 7 post-surgery (Figure 6C). The saline and LHM@PPs groups exhibited weak VEGF-positive signals and low fluorescence intensity, indicating limited angiogenic capacity. The HHM@VP group showed anti-scarring effects in the later stage but delayed healing due to insufficient early anti-inflammatory action, and VEGF expression was suboptimal.

Notably, the BHM@VP@PPs + L group exhibited markedly elevated VEGF fluorescence signals, accompanied by expanded positive covered areas that extensively covered the epidermis at wound margins and newly formed granulation tissue. These findings indicate that a robust angiogenic foundation was established during the early stage of wound healing. Quantitative analysis using ImageJ further confirmed that VEGF expression levels in this group were 15-fold higher than those in the control group (Figure 6D), while collagen deposition was 3-fold higher (Figure 6E). Moreover, the number of newly formed hair follicles was 60-fold greater than that in the control group (Figure 6F). Based on these results, it is inferred that in the BHM@VP@PPs + L group, the PBA-PEI@siRNA (TNF-α) complex loaded in the lower LHM@PPs layer was efficiently delivered to macrophages at the wound site. By inhibiting TNF-α mRNA expression, the complex blocked aberrant inflammatory signaling cascades and reduced pro-inflammatory cytokine levels. This early modulation of the immune microenvironment created favorable conditions for the expression of pro-angiogenic factors such as VEGF, facilitating a smooth transition from the inflammatory phase to the proliferative phase and achieving rapid early healing.

#### 2.8.4. Scar Assessment

On postoperative day 28, the wound in the BHM@VP@PPs + L group showed almost no visible scar, with smooth skin and a color close to normal, indicating good healing quality (Figure 7A). In contrast, the saline, LHM@PPs, and HHM@VP groups all exhibited significant residual scarring, accompanied by varying degrees of hyperplasia or atrophy and disrupted skin texture. COL I/COL III immunofluorescence staining (Figure 7A,B) revealed that in the BHM@VP@PPs + L group, type I collagen (red) expression was significantly lower than in the control (without VP) and LHM@PPs groups, whereas type III collagen (green) expression showed the opposite trend. The type I/III collagen ratio was markedly reduced in this group; ImageJ quantification showed a ratio of 5.8 in the control group compared with only 0.5 in the treatment group.

Sirius Red staining was performed on wound tissue collected on day 28, and the distribution of type I (yellow) and type III (green) collagen was observed under a polarized light microscope (Figure 7A,C). The saline and LHM@PPs groups were dominated by type I collagen, with type III collagen almost invisible. In contrast, the BHM@VP@PPs + L group was dominated by type III collagen. ImageJ quantification showed that the type I/III collagen ratio (0.75) in the treatment group was significantly lower than that in the saline group (1.59). Further analysis of the relationship between treatment strategy and scar formation revealed that LHM@PPs intervened only during the inflammatory phase and did not regulate the proliferative phase, leading to uncontrolled fibroblast proliferation, unbalanced collagen metabolism, and larger scars. Although HHM@VP inhibited fibroblast transformation during the proliferative phase, it lacked early anti-inflammatory effects, resulting in delayed healing and noticeable scars. In contrast, the combination of BHM@VP@PPs and PDT released siRNA for targeted anti-inflammation in the inflammatory phase and released VP to inhibit the YAP/TAZ pathway in the proliferative phase, while PDT simultaneously produced ROS for antibacterial effects. The combined stage-matched intervention of these three mechanisms made the wound healing process more closely resemble normal biological repair, significantly reducing scar formation.

#### 2.8.5. In Vivo Biosafety

To evaluate the in vivo biosafety of the BHM@VP@PPs combined with PDT, mice from each group were euthanized on day 28 post-treatment. Major organs (heart, liver, spleen, lungs, and kidneys) were collected, fixed, embedded, sectioned, and stained with H&E. Histopathological changes were observed under a light microscope (Figure S17). No significant organ damage was observed, indicating good biocompatibility and long-term safety.

### 2.9. Transcriptomic Analysis

To investigate the mechanism of action of the BHM@VP@PPs combined with PDT, wound tissue was collected on day 28 for transcriptomic sequencing (Figure S18). Compared with the control group, a total of 1063 differentially expressed genes were identified, of which 549 were upregulated and 514 were downregulated. The balanced number of up- and down-regulated genes indicates that this treatment exerts a broad and systematic regulatory effect on wound transcription. These differentially expressed genes were primarily enriched in biological processes such as inflammatory responses, immune regulation, cell proliferation and differentiation, and extracellular matrix remodeling, providing important transcriptomic evidence for further elucidating the network mechanisms by which this bilayer hydrogel temporally regulates wound healing.

KEGG enrichment analysis (Figure 7D) showed that the differentially expressed genes were mainly enriched in pathways including IL-17, NF-κB, JAK-STAT, HIF-1, TGF-β, and Hippo. These pathways play key roles in wound healing; notably, the TGF-β and Hippo pathways are involved in fibroblast activation, collagen deposition, and fibrosis, and are closely associated with scar formation. GO functional enrichment analysis (Figure 7E) indicated that the differentially expressed genes were significantly enriched in biological processes such as cellular response to TGF-β stimulation, extracellular matrix organization, collagen fiber organization, and regulation of fibroblast proliferation, further validating the modulatory effect of VP on the YAP/TAZ pathway. In summary, transcriptomic analysis revealed the complex network mechanism by which the combined BHM@VP@PPs and PDT promote scar-minimizing healing through the synergistic regulation of inflammation, immunity, proliferation, and fibrosis via multiple pathways.

However, due to the absence of a simultaneous-release control group, the current study cannot decouple the specific benefits of sequential drug delivery from the general effects of combined drug administration. Future work incorporating simultaneous-release hydrogel control is therefore warranted to further validate the functional superiority of the sequential delivery strategy.

## 3. Conclusions

In conclusion, an HM bilayer hydrogel (BHM@VP@PPs) was developed, in which the lower layer was loaded with PBA-PEI@siRNA (TNF-α) nanocomposites and the upper layer was loaded with the small-molecule YAP inhibitor VP, for scar-minimizing healing of deep second-degree burn wounds. By tuning the degradation rates of the two layers, the hydrogel sequentially releases siRNA and VP to promote macrophage polarization during the inflammatory phase to modulate the immune microenvironment and regulate the fibroblast-to-myofibroblast transition during the proliferative phase. In addition, VP exerts antibacterial effects under PDT. Our results demonstrate that this BHM@VP@PPs bilayer hydrogel enables scar-minimizing healing of chronic deep second-degree burn wounds on the dorsal skin of mice. Moreover, the hydrogel can conform to wounds of any shape, making it suitable for treating irregular wounds encountered in clinical practice. This system therefore holds high potential for clinical translation and provides a novel therapeutic strategy for the clinical management of chronic wounds such as deep second-degree burns. Nevertheless, its antimicrobial efficacy has only been validated against *S. aureus* and *E. coli*, and its performance against biofilm infections remains unevaluated. Future work will focus on developing microenvironment-responsive drug release mechanisms tailored to the conditions of infected wounds. Burn wound models with bacterial infection should also be adopted to comprehensively assess the anti-infective and anti-scarring effects of this hydrogel system. Furthermore, this sequential intervention strategy can be extended to other types of chronically infected wounds, such as diabetic foot ulcers, thereby advancing the clinical translation of integrated wound dressings.

## 4. Materials and Methods

### 4.1. Materials

Hyaluronic acid was purchased [molecular weight (MW) = 100 kDa, 98%], Verteporfin (VP, purity ≥ 98.15%) was purchased from Med Chem Express (Monmouth Junction, NJ, USA), Methacrylic anhydride (94%, containing 0.2% Toprol stabilizer), and branched polyethyleneimine (MW = 25 kDa) were purchased from Sigma-Aldrich (Darmstadt, Germany), 4-(bromomethyl)phenylboronic acid (purity ≥ 98%) was purchased from Macklin (Beijing, China), and the DPBF probe (purity ≥ 99%), 2′,7′-Dichlorodihydrofluorescein diacetate (DCFH-DA) probe were purchased from Beyotime Biotechnology (Shanghai, China), while siRNA (TNF-α), and fluorescently labeled siRNA were purchased from Gene Pharma (Suzhou, China).

### 4.2. Synthesis and Characterization of PBA-PEI

A total of 100 mg of PBA was placed into a round-bottom flask, and 5 mL of methanol was added. The mixture was stirred until complete dissolution. Separately, 300 mg of PEI was dissolved in 3 mL of methanol. The PEI solution was then added dropwise to the PBA solution in an oil bath maintained at 60 °C. After reacting for 24 h, the crude product was preliminarily purified by precipitation with cold ether. The precipitate was collected by centrifugation, washed three times with cold ether, and then dried under vacuum overnight, yielding a pale-yellow powder. Subsequently, the powder was dissolved in deionized water and dialyzed against deionized water for two days (MWCO: 3 kDa). Finally, the dialyzed solution was freeze-dried to obtain the purified PBA-PEI product.

Fourier transform infrared (FT-IR) spectra of the dried PBA and PBA-PEI samples were recorded in the range of 400–4000 cm^−1^ using a Nicolet iS20 spectrometer (Xi’an, China). PBA, PEI, and PBA–PEI were separately dissolved in heavy water, and the ^1^H NMR spectra were acquired using a Bruker Avance NEO 400 MHz spectrometer (Hangzhou, China) with a scanning range of 0–9 ppm. Elemental analysis (EA) of the resulting PBA-PEI powder was performed using an Elementar Unicube analyzer (Hangzhou, China) to determine the contents of carbon (C), hydrogen (H), nitrogen (N), and oxygen (O).

### 4.3. Synthesis of PBA-PEI@siRNA Nanocomposites

An appropriate amount of PBA-PEI powder was weighed and dissolved in DEPC water to prepare a stock solution of 1 mg/mL. TNF-α siRNA dry powder (1 OD) was reconstituted by low-speed centrifugation, followed by the addition of 100 μL of DEPC-treated water to obtain a 25 μM solution. Based on the grafting ratio determined by elemental analysis, the mass ratio of PBA-PEI to siRNA was calculated. The PBA-PEI solution and the siRNA solution were then mixed at various N/P ratios (M = nitrogen/phosphate mass ratios). The mixtures were incubated at room temperature for 30 min to prepare the PBA-PEI@siRNA nanocomposites. The particle size and zeta potential of the nanocomposites at each N/P ratio were measured at room temperature using an Anton Paar (Vienna, Austria) Litesizer DLS 501 dynamic light scattering instrument to determine the optimal N/P ratio. Furthermore, the as-prepared PBA-PEI@siRNA nanocomposites were uniformly dispersed in DEPC water. After appropriate dilution with DEPC water, the mixture was subjected to ultrasonic dispersion for 10 min. A 10 μL droplet of the nanocomposite dispersion was deposited onto a carbon-coated copper grid. Following air drying, high-resolution images of the nanocomposites were acquired using a transmission electron microscope (Xi’an, China) to characterize their microstructure and particle size.

### 4.4. Binding Capacity and Serum Stability of Nanocomposites

Agarose powder (3 g) was dissolved in 100 mL of TAE buffer and heated in a microwave until completely dissolved to prepare a 3% (*w*/*v*) agarose gel. Gel Red nucleic acid stain was added, mixed thoroughly, and the gel was cast. PBA-PEI@siRNA nanocomposites prepared at different N/P ratios were mixed with loading buffer, and equal volumes of samples were loaded onto the gel. Electrophoresis was performed at 90 V for 40 min. Free siRNA was used as the control group.

To evaluate serum stability, PBA-PEI@siRNA nanocomposites with an N/P ratio of 7.5 were prepared. Each sample was diluted to 50 μL with culture medium containing 10% or 50% fetal bovine serum (FBS), respectively, and incubated at 37 °C for 1, 3, 6, 9, 12, and 24 h. The samples were mixed with loading buffer containing 2% SDS to dissociate the complexes. A free siRNA control group was also established; the free siRNA samples were incubated under the same conditions. All samples were subjected to agarose gel electrophoresis simultaneously under the same conditions as described above. Following electrophoresis, images were captured using a gel imaging system, and the band intensity (grayscale values) was analyzed using ImageJ software to assess serum stability. The experiment was repeated three times.

### 4.5. Synthesis of HM

HM (Methacrylate hyaluronic acid) was synthesized by reacting methacrylic anhydride (MA) with hyaluronic acid (HA) in an aqueous solution. Briefly, 1 g of HA was dissolved in 100 mL of deionized water to obtain a 1% (*w*/*v*) HA solution. Separately, 1 mL of MA was dispersed in 100 mL of deionized water under magnetic stirring to prepare a 1% (*v*/*v*) MA solution. The MA solution was then added to the HA solution, and the pH of the mixture was adjusted to 8–10 by dropwise addition of 3 M NaOH. The reaction was carried out in the dark in an ice bath for 24 h. Subsequently, the reaction mixture was dialyzed against deionized water for 3 days. Finally, the dialyzed solution was freeze-dried to obtain the purified HM powder.

The FT-IR spectra of the HM powder were measured in the range of 400–4000 cm^−1^ using a Nicolet iS20 spectrometer (Hangzhou, China). Additionally, the ^1^H NMR spectra of the HM powder were measured in the range of 0–9 ppm using an Ascend TM 600 MHz spectrometer (Hangzhou, China).

### 4.6. Cell Compatibility Assay

RAW 264.7 cells were seeded into 96-well plates at a density of 5 × 10^3^ cells per well and cultured in an incubator for 24 h to allow full adherence. Subsequently, the cells were treated with 100 μL of HM hydrogel extract (0.1 mg/mL) or with different concentrations of PBA-PEI solution (0–10 μg/mL) for 24 h. After treatment, the supernatant was aspirated, and 110 μL of culture medium containing 10% CCK-8 was added to each well. A blank control group was also prepared. Following incubation in the dark for 2 h, the optical density (OD) at 450 nm was measured using a multifunctional microplate reader. The cell viability was calculated using the following formula:

$$\mathrm{Cell}\;\mathrm{viability}\;(\%)=\frac{\left({\mathrm{OD}}_{Test}-{\mathrm{OD}}_{Blank}\right)}{\left({\mathrm{OD}}_{Control}-{\mathrm{OD}}_{Blank}\right)}\times 100\%$$

where OD_Test_ represents the absorbance of cells treated with HM hydrogel extract or PBA-PEI solution, OD_Control_ represents the absorbance of untreated cells (negative control), and OD_Blank_ represents the absorbance of the blank control group.

Hemolysis assays were performed to evaluate the hemocompatibility of each hydrogel group. Fresh blood was collected from healthy rabbits. After centrifugation, the supernatant was discarded, and the red blood cells were resuspended in phosphate-buffered saline (PBS). This washing procedure was repeated three times, and the final RBC pellet was collected. The RBCs were then diluted with PBS to a final concentration of 5% (*v*/*v* %). Each hydrogel (LHM, LHM@PPs, HHM, HHM@VP, and BHM@VP@PPs) was sterilized and subsequently incubated at 37 °C for 24 h to obtain the respective hydrogel extracts. Equal volumes of the hydrogel extract and the 5% RBC suspension were mixed. Deionized water (dd H_2_O) was used as the positive control, and PBS as the negative control. All samples were incubated at 37 °C for 1 h, followed by centrifugation. After centrifugation, taking photographs, the supernatant from each sample was collected, and transferred to a 96-well plate. The absorbance at 545 nm was measured using a multifunctional microplate reader. The hemolysis rate was calculated using the following formula:

$$\mathrm{Hemolysis}\;\mathrm{rate}\;(\%)\;=\frac{{\mathrm{OD}}_{Sample}-{\mathrm{OD}}_{PBS}}{{\mathrm{OD}}_{ddH2O}-{\mathrm{OD}}_{PBS}}\;\times \;100\%$$

where OD_Sample_ is the absorbance of the supernatant from the hydrogel-treated sample, OD_PBS_ is the absorbance of the negative control, and OD_ddH2O_ is the absorbance of the positive control.

### 4.7. SEM of BHM@VP@PPs Hydrogels

The fabrication procedure of the bilayer hydrogel was carried out as follows: First, the bottom hydrogel layer was prepared by blending pre-synthesized PBA-PEI@siRNA (TNF-α) nanocomposite (containing 2 µg siRNA) with 2% (*w*/*v*) HM solution and 1% (*w*/*v*) photoinitiated 2-Hydroxy-4′-(2-hydroxyethoxy)-2-methylpropiophenone (Ir2959) solution. The three components were fully homogenized via vortex mixing. After 10 s of UV irradiation (365 nm), the bottom hydrogel layer was formed. Subsequently, the top hydrogel layer was fabricated directly on the surface of the pre-formed bottom layer. Verteporfin (VP, 5 µg) was mixed with 8% (*w*/*v*) HM solution and 1% (*w*/*v*) Ir2959 solution, followed by thorough vortex homogenization. The resulting mixture was cast onto the bottom hydrogel and exposed to 365 nm UV light for 5 s to trigger gelation. This process enables in situ interlayer crosslinking to form the bilayer hydrogel. Subsequently, the cross-sectional microstructure was characterized by scanning electron microscopy (SEM). Briefly, the as-prepared bilayer hydrogel was rapidly frozen in liquid nitrogen and fractured to expose its cross-section. After freeze-drying, the sample undergoes gold sputtering, and its microstructure was imaged using a scanning electron microscope (Shanghai, China).

### 4.8. Swelling Behaviors of Hydrogels

The swelling test was performed as follows: Hydrogels from different groups were fully immersed in PBS. At predetermined time intervals, each hydrogel was removed, the excess surface water was gently wiped off, and the residual weight was recorded. The swelling equilibrium was reached when the weight remained essentially constant. Swelling ratio was calculated based on the mass difference between the wet and initial states to quantify the water absorption capacity of the hydrogels. The swelling ratio was calculated using the following formula:

$$\mathrm{Swelling}\;\mathrm{ratio}\;(\%)\;=\frac{W_{T}-W_{0}}{W_{0}}\times 100\%$$

where W_0_ is the initial weight of hydrogel, and W_T_ is the weight of the hydrogel at a given time point during swelling in PBS.

### 4.9. Degradation Behaviors of Hydrogel

Once swelling equilibrium was reached, the degradation of the hydrogels was evaluated using a method analogous to that of the swelling test. Briefly, the hydrogels were incubated in PBS at 37 °C. At predetermined time intervals, the hydrogel was removed, gently blotted to remove surface water, and weighed. The degradation rate was calculated using the following formula:

$$\mathrm{Mass}\;\mathrm{remaining}\;(\%)\;=\frac{W_{B}-W_{t}}{W_{B}}\times 100\%$$

where W_B_ is the initial weight of the hydrogel at the swelling equilibrium, and W_t_ is the weight of the hydrogel at a given time point during the degradation period.

### 4.10. Rheological Properties of Hydrogel

The compressive mechanical properties of single-layer, double-layer, and upper-lower layer hydrogels were evaluated using a Model 3354 electronic universal testing machine (Xi’an, China). Hydrogel samples were prepared as cylinders with a diameter of 10 mm and a thickness of 5 mm and then placed on the compression testing platform. The compression rate was set to 1 mm/min, and the test was terminated when the strain reached 80%. The stress–strain data during compression were recorded, and a curve was plotted with strain on the x-axis and stress on the y-axis.

An Anton Paar (Vienna, Austria) was used to measure the storage modulus ($G^{\prime }$) and loss modulus ($G^{\prime \prime }$) of LHM, BHM, and HHM hydrogels, as well as their gelation thresholds under UV irradiation. Hydrogel samples were prepared as cylinders with a thickness of 2 mm and a diameter of 25 mm, placed on the rheometer’s optical testing platform, and measured using a parallel-plate rotor of 25 mm diameter. The rheological properties were evaluated through the following tests:

Angular frequency sweep: performed under a constant strain of 1% over an angular frequency range of 0.1–100 rad/s.

Strain sweep: conducted at a constant angular frequency of 10 rad/s over a strain range of 0.01–1000%.

The gelation threshold was determined at a constant temperature of 37 °C. The gelation critical point was defined as the moment when the storage modulus ($G^{\prime }$) first became equal to or greater than the loss modulus ($G^{\prime \prime }$). The storage modulus measured after complete gelation was recorded as the final elastic modulus. Precursor solutions of the upper-layer, lower-layer, and bilayer hydrogels were each separately taken and uniformly spread onto the optical detection platform of the rheometer. The shear strain was set to 1%. Because the gelation process occurred relatively rapidly, one data point was collected every 0.5 s. UV irradiation was applied 12 s after the start of the measurement. The changes in the storage modulus and loss modulus during the gelation process under two different conditions were investigated: (1) the UV light source was removed after 12 s of irradiation, and (2) irradiation was continued until the end of the experiment.

### 4.11. Release of siRNA and VP

VP and FAM-labeled siRNA were encapsulated in the upper and lower layers of the bilayer hydrogel, respectively. The bilayer hydrogel samples were incubated in the dark, and eluates were collected at predetermined time points. An aliquot of 200 μL of the eluate was transferred to a 96-well plate. A multi-mode microplate reader (Xi’an, China) was used to measure the fluorescence intensity at 490 nm and absorbance at 689 nm for quantitative analysis of siRNA and VP release.

### 4.12. Types of ROS Produced by VP

#### 4.12.1. Fluorescence Assay

To evaluate the ROS-generating capacity of VP upon photodynamic therapy (PDT), a 2′,7′-dichlorodihydrofluorescein diacetate (DCFH-DA) probe was employed. Briefly, 2 mL of DCFH-DA (1 mM) was added to 8 mL of NaOH solution (10 mM), and the mixture was incubated at room temperature for 30 min to hydrolyze DCFH-DA into DCFH. Then, 40 mL of PBS (pH 7.4) was added to stop the reaction, yielding a DCFH stock solution. Subsequently, 3 mL of the DCFH stock solution was mixed with 100 μg of VP. The mixture was irradiated with red light at 660 nm (25 mW/cm^2^) for various time intervals (0, 1, 3, 6, 10, 15, 21, and 30 min). At each point, the fluorescence intensity at 525 nm was measured using a fluorescence spectrophotometer. A control group containing only DCFH solution (without VP) was also prepared to eliminate any background interference.

#### 4.12.2. EPR Assay

Detection of singlet oxygen (^1^O_2_): A total of 1 mg of VP was weighed and dispersed in 1 mL of deionized water. After sonication, the singlet oxygen scavenger TEMP (2,2,6,6-tetramethyl-4-piperidone hydrochloride) and a catalyst were added, and the mixture was thoroughly mixed. The EPR signal of the unirradiated sample (ground state) was recorded. The remaining samples were then irradiated with red light at 660 nm for 10 min, and the EPR signals were measured again.

Detection of superoxide anion (·O_2_^−^): 1 mg of VP was dispersed in 1 mL of ethanol, followed by the addition of the scavenger DMPO (5,5-Dimethyl-1-pyrroline N-oxide) and a catalyst. The EPR signals were measured both before and after irradiation using the same irradiation conditions as described above.

#### 4.12.3. Chemical Method Using DPBF

The generation of ^1^O_2_ was further confirmed using a chemical method with DPBF (1,3-Diphenylisobenzofuran). An appropriate amount of VP powder was dissolved in ethanol (containing 1% DMSO) to prepare a 1 mM stock solution. DPBF was dissolved in anhydrous ethanol to obtain a 10 mM stock solution. Both stock solutions were then diluted with PBS (pH 7.4) to the required working concentrations. A working solution containing only VP was prepared, and its full-wavelength absorption spectrum was recorded. Then, an appropriate volume of the DPBF stock solution was added to the VP working solution and mixed thoroughly. The absorption spectrum of the mixture in the range of 300–500 nm was measured. The mixture was subsequently irradiated with a 660 nm red light source at different power densities (0, 5, 25, and 50 mW/cm^2^). At various time points (1, 3, 8, and 15 min), aliquots were taken, and the total fluorescence intensity (alternatively, the absorbance) at 400–500 nm was measured. All operations were performed in the dark to avoid environmental quenching of DPBF fluorescence. In addition, the full-wavelength spectrum (300–800 nm) of the mixed solution was recorded under the above irradiation conditions to evaluate the photostability of VP after exposure to 660 nm light.

### 4.13. Transfection and Gene Silencing Efficiency of PBA-PEI@siRNA

#### 4.13.1. Fluorescence Observation

RAW 264.7 cells were seeded into 24-well plates at a density of 2 × 10^4^ cells per well. After 24 h of culture, the medium was replaced with serum-free DMEM containing PBA-PEI@siRNA nanocomposites at different N/P ratios (the final concentration of FAM-labeled siRNA was 50 nM), and the cells were incubated for 6 h. The cells were then washed three times with PBS, and images were captured under a fluorescence microscope at randomly selected fields of view. Free FAM-siRNA was used as a control. Fluorescence intensity was semiquantitatively analyzed using ImageJ software. The experiment was performed in triplicate.

#### 4.13.2. Flow Fluorescence Intensity Analysis

To quantitatively evaluate the cellular uptake efficiency, RAW 264.7 cells (1 × 10^5^ cells per well) were seeded into 6-well plates. After 24 h of culture, the cells were incubated for 6 h with serum-free DMEM containing either free FAM-siRNA or PBA-PEI@siRNA nanocomposites at various N/P ratios (final FAM-siRNA concentration: 50 nM). The culture medium was then discarded, and the cells were washed three times with PBS. Subsequently, 1 mL of PBS was added to each well, and the cells were detached using a cell scraper and transferred into centrifuge tubes. After centrifugation, the supernatant was removed, and the cell pellets were washed three times with PBS. Finally, the cells were resuspended in 200 μL of PBS. Fluorescence intensity was measured using a flow cytometer, and the mean fluorescence intensity (MFI) was quantified using ImageJ software. The experiment was repeated three times.

#### 4.13.3. Gene Silencing Efficiency Evaluation

The TNF-α protein concentration in the culture supernatant of RAW 264.7 cells was measured using an enzyme-linked immunosorbent assay (ELISA) kit according to the manufacturer’s instructions. Meanwhile, the cells were collected, and the TNF-α mRNA expression levels were determined by reverse transcription-quantitative polymerase chain reaction (RT-qPCR) to assess the gene silencing efficiency. The experimental groups included: a blank control group; an LPS-stimulated group; a PBA-PEI@siRNA (NC) complex group (negative control siRNA) and the three different siRNA sequences were each conjugated with the PBA-PEI cationic polymer to form nanocomposites, which were designated as PBA-PEI@siRNA-1 (559), PBA-PEI@siRNA-2 (644), and PBA-PEI@siRNA-3 (729). The siRNA sequences targeting the TNF-α gene, the negative control (NC) siRNA sequence, and the positive control (GAPDH) siRNA sequences were synthesized by Gene Pharma. The sequences are listed in Table S2.

Based on the threshold cycle (Ct) values obtained from each experimental group, the relative expression levels of TNF-α mRNA were calculated using the 2^−ΔΔCt^ method. TNF-α was used as the target gene, and GAPDH was used as the internal reference. The primer sequences for both genes are listed in Table S3. The experiment was repeated three times.

### 4.14. Evaluation of Anti-Inflammatory Effects of Hydrogels

#### 4.14.1. Cytokine Secretion Analysis by ELISA

RAW 264.7 cells were seeded into 6-well plates at a density of 1 × 10^5^ cells per well. After 24 h of culture, the medium was discarded, and the cells were washed three times with PBS. The cells were then treated with pre-warmed (37 °C) serum-free DMEM containing either the PBA-PEI@siRNA nanocomposite, LHM@PPs extract, or LHM extract, respectively. The culture plates were gently swirled to ensure even mixing and then incubated for 6 h. After incubation, the medium was replaced with complete DMEM, and the cells were further cultured for 48 h. The culture medium was then removed, and the cells were washed once with PBS. Subsequently, the cells were stimulated with complete DMEM containing 500 ng/mL lipopolysaccharide (LPS) for 6 h. An untreated blank control group (no LPS stimulation) was also established. After stimulation, the cell culture supernatant was collected into 1.5 mL centrifuge tubes and centrifuged to remove cell debris. The clear supernatant was transferred to new tubes. The protein levels of tumor necrosis factor-alpha (TNF-α), interleukin-6 (IL-6), interleukin-10 (IL-10), and transforming growth factor-beta (TGF-β) in the supernatant were measured using enzyme-linked immunosorbent assay (ELISA) kits according to the manufacturers’ instructions.

#### 4.14.2. Flow Cytometric Immunofluorescence Analysis

To further evaluate the anti-inflammatory effects of the LHM@PPs hydrogel, the expression of surface markers CD86 (FITC, green, pro-inflammatory phenotype M1) and CD206 (APC, red, anti-inflammatory phenotype M2) on RAW 264.7 cells was detected by flow cytometry. Cells were treated in the same manner as in the ELISA experiment. After stimulation with LPS, the cells were harvested, stained with the corresponding antibodies (CD86-FITC and CD206-APC) following the manufacturers’ protocols, and analyzed using a flow cytometer.

#### 4.14.3. Immunofluorescence Staining Analysis

Immunofluorescence staining was performed to visually observe the changes in macrophage phenotype. Sterilized cell smears were placed into 6-well plates. Macrophages were treated as described above (same as for ELISA). After the treatment, the cell smears were incubated with anti-CD86 (green) and anti-CD206 (red) antibodies, respectively. Cell nuclei were counterstained with DAPI (blue). Finally, the samples were observed and photographed under a fluorescence microscope, and the expression of each marker was analyzed.

### 4.15. ROS Production by VP In Vitro

#### 4.15.1. Microscopy Observation

3T3 cells were seeded into 24-well plates at a density of 2 × 10^4^ cells per well. After 24 h of culture, the cells were treated with either HHM hydrogel extract, free VP drug, or HHM@VP hydrogel extract, respectively. A blank control group was also established. The plates were gently shaken to ensure even mixing of the contents. After incubation in the dark for 6 h, the cells were incubated with the DCFH-DA probe (10 μM diluted in PBS) for an additional 30 min in the dark. Subsequently, 660 nm photodynamic therapy (PDT) treatment was performed for 10 min to induce ROS production. The cells were then washed three times with PBS, and the nuclei were stained with Hoechst 33342 at room temperature for 10 min, followed by three additional washes with PBS. Intracellular ROS levels were observed under an inverted fluorescence microscope. Random images were captured, and the fluorescence intensity was semi-quantitatively analyzed using ImageJ software.

#### 4.15.2. Flow Cytometry Analysis

To further validate the ROS-generating ability of the HHM@VP hydrogels under photodynamic therapy (PDT), flow cytometry was performed on the treated 3T3 cells. After the same treatment procedure as described above, the cells were detached by adding 1 mL of trypsin-EDTA solution and collected into 1.5 mL centrifuge tubes. After centrifugation, the supernatant was discarded, and the cell pellets were washed three times with PBS. Finally, the cells were resuspended in 300 μL of PBS, and the fluorescence intensity was measured using a flow cytometer.

### 4.16. In Vitro Antimicrobial Efficacy of Hydrogels

The antimicrobial activity of the drug-loaded hydrogel under photodynamic therapy (PDT) was evaluated against *Staphylococcus aureus* (*S. aureus*) and *Escherichia coli* (*E. coli*). Five experimental groups were established: (1) Control group (untreated bacterial suspension), (2) Laser group (660 nm light irradiation), (3) HHM + L group (hydrogel and 660 nm light irradiation), (4) VP + L group (VP and 660 nm light irradiation), and (5) HHM@VP + L group (VP-loaded hydrogel and 660 nm light irradiation). Samples from each group were added to a bacterial suspension of *S. aureus* and *E. coli* at a concentration of 1 × 10^5^ colony-forming units per milliliter (CFU/mL). After PDT treatment for 10 min, the optical density (OD) of each bacterial suspension was measured at a wavelength of 600 nm using a multifunctional microplate reader. An appropriate volume of the bacterial suspension was then serially diluted, and aliquots were spread onto solid agar plates. The plates were incubated at 37 °C for 24 h, after which photographs were taken to record colony growth. For morphological observation, 10 μL of the PDT-treated bacterial suspension was placed onto a glass slide and fixed overnight at room temperature with 4% paraformaldehyde. The samples were then dehydrated sequentially in 25%, 50%, 75%, and 100% ethanol for 30 min each, air-dried, and sputter-coated with gold. Bacterial morphology was observed using SEM.

Western blotting was performed to detect the expression of the anti-scarring-related protein YAP (Yes-associated protein) to evaluate the anti-scarring function of the small-molecule inhibitor VP. 3T3 cells were seeded into 12-well plates at a density of 1 × 10^4^ cells per well. After 24 h of culture, the cells were treated with different concentrations of VP (0, 1, 2, and 5 μg/mL) and incubated for an additional 24 h. Total proteins were extracted using RIPA lysis buffer (containing protease and phosphatase inhibitors) and quantified using the BCA method. Equal amounts of protein were loaded onto an SDS-polyacrylamide gel, separated by SDS-PAGE, and transferred onto a polyvinylidene difluoride (PVDF) membrane. The membrane was blocked with a blocking solution containing 10% (*w*/*v*) non-fat dry milk in TBST for 2 h at room temperature. Subsequently, the membrane was incubated overnight at 4 °C with diluted primary antibodies against YAP, α-SMA (alpha-smooth muscle actin), and GAPDH. After incubation, the membrane was washed three times with TBST followed by incubation with secondary antibody at room temperature for 1 h. After thorough washing with TBST, the protein bands were visualized using a chemiluminescence (ECL) detection kit. Band intensities were quantified using ImageJ software. The experiment was repeated three times.

### 4.17. Deep Second-Degree Burn Models

All animal procedures were approved by the Animal Ethics Committee of Northwest University (Approval No. NWU-IACUC-20251109M). To evaluate the repair effect of the BHM@VP@PPs bilayer hydrogel combined with PDT on deep second-degree burn wounds, male C57BL/6 mice aged 6–7 weeks were used. The animals were acclimatized to the housing conditions for one week prior to the experiment. One day before wound modeling, the mice were anesthetized with 3–5% isoflurane. The dorsal hair was shaved, and the skin was cleaned with sterile saline. On the day of modeling, a deep second-degree burn was induced using a thermostatic burn device. A metal rod with a diameter of 8 mm was preheated to 85 °C and allowed to contact the shaved dorsal skin for 15 s under its own weight, without additional pressure. Immediately after successful burn induction, 1 mL of Ringer’s lactate solution was administered subcutaneously to prevent dehydration. At 24 h post-burn, the wound was debrided, and the mice were randomly divided into six groups: (1) Control group (saline), (2) LHM@PPs group, (3) HHM@VP group, (4) HHM@VP and PDT treatment group (HHM@VP + L), (5) BHM@VP@PPs group, and (6) BHM@VP@PPs and PDT treatment group (BHM@VP@PPs + L). The corresponding hydrogel or saline was applied to each wound. Digital photographs were taken, and wound healing progression was recorded on days 0, 3, 7,15 and 21 post-treatment. The wound area and healing rates at each time point were calculated by image analysis using ImageJ software.

### 4.18. Histopathological Analysis

Histological evaluation of deep second-degree burn wounds was performed using hematoxylin and eosin (H&E) staining and Masson’s trichrome staining. Mice were euthanized on postoperative days 7, 15, and 21, respectively. Full-thickness skin tissue samples from the wound site were harvested and fixed in 4% paraformaldehyde. After rinsing with PBS, the tissues were dehydrated through a graded ethanol series, cleared in xylene, embedded in paraffin, sectioned, and mounted onto glass slides. The sections were then deparaffinized, rehydrated, and stained with H&E or Masson’s trichrome according to standard protocols. Stained sections were observed under a light microscope, and images were captured. To assess the long-term in vivo biosafety of the hydrogel material, major organs (heart, liver, spleen, lung, and kidneys) were collected from the mice on postoperative day 28. The organs were fixed, embedded, sectioned, and stained with H&E following the same procedure as described above. Excess stain was removed with deionized water. After mounting and drying, the histological structure and morphology of each organ were examined under a microscope.

### 4.19. Fluorescent Immunohistochemistry Analysis

Skin tissue samples were collected from burn wounds on postoperative days 7 and 28. After fixation with 4% paraformaldehyde, the tissues were embedded in paraffin and sectioned using a microtome. The sections were then blocked with a PBS solution containing 1% (*w*/*v*) bovine serum albumin (BSA) for 1 h at room temperature. Subsequently, the sections were incubated overnight at 4 °C with the corresponding primary antibodies. After incubation, the sections were washed three times with PBS to remove unbound primary antibodies. Next, the sections were incubated with fluorescently labeled secondary antibodies for 1 h at room temperature in the dark. Finally, cell nuclei were counterstained with DAPI for 5–10 min. After mounting with an anti-fade mounting medium, the stained sections were observed and photographed under a fluorescence microscope. Tissues collected on day 7 were used to assess neovascularization by staining for vascular endothelial growth factor (VEGF). Tissues collected on day 28 were stained for collagen type I (COL I) and collagen type III (COL III), and the COL I/COL III ratio was calculated to evaluate scar formation in the different treatment groups. Images were captured for quantitative statistical analysis.

### 4.20. Sirius Red Staining Analysis

Skin tissue samples were collected from the wound site on postoperative day 28, fixed in 4% paraformaldehyde, and embedded in paraffin. Tissue sections were prepared using a microtome. After deparaffinization, the paraffin sections were sequentially rehydrated through a graded ethanol series and then washed with distilled water. The sections were stained with Sirius Red solution for 60 min at room temperature. Following staining, the sections were dehydrated, cleared, and mounted with a mounting medium. The stained sections were examined under both a light microscope and a polarizing microscope, and images were captured for subsequent quantitative analysis. Under polarized light, type I collagen appears bright red or orange, whereas type III collagen appears greenish, allowing for semi-quantitative evaluation of collagen composition.

### 4.21. Transcriptomic Methodology

Total RNA was extracted from day-28 wound tissues for RNA-seq analysis. Differential expression analysis was performed using the R Bioconductor 3.2 package “edgeR” or “DESeq2”. KEGG pathway and GO enrichment analyses were carried out with The R package “ClusterProfiler”.

### 4.22. Statistical Analysis

The results of statistical analysis were expressed as the mean ± standard deviation. The experimental results were analyzed and processed using ImageJ 1.52a software. All data are expressed as mean ± standard deviation (SD). One-way ANOVA followed by Tukey’s multiple-comparisons test was used for multi-group comparison in GraphPad Prism 10.1.2 software. *p* < 0.05 was defined as statistically significant, with statistical significance indicated by: * *p* < 0.05, ** *p* < 0.01, *** *p* < 0.001; ns = no significance, *p* > 0.05. All in vitro experiments were independently repeated at least three times.

## Figures and Tables

**Figure 1:**
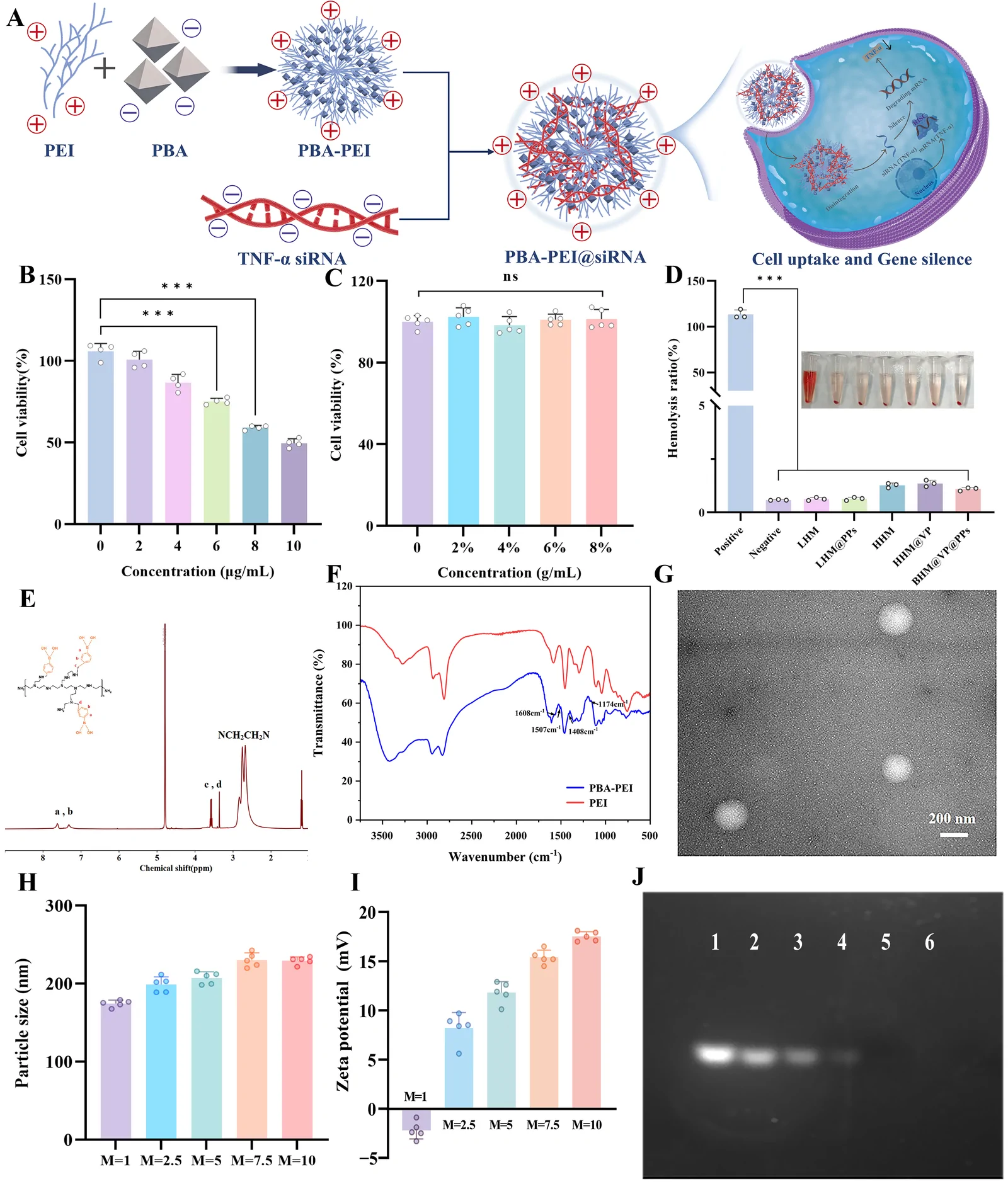
Preparation and characterization of PBA-PEI and PBA-PEI@siRNA nanocomposites. (**A**) Schematic illustration of the synthesis of PBA-PEI@siRNA and its cellular internalization via endocytosis. (**B**) Cell viability rates of RAW 264.7 cells treated with different concentrations of PBA-PEI. (**C**) Cell viability rates of RAW 264.7 cells treated with different concentrations of HM hydrogel extracts. (**D**) Visual assessment of hemolytic activity of different hydrogel groups, positive and negative controls, and clotting properties of the hydrogels. (**E**) ^1^H NMR spectra of PBA-PEI. (**F**) FT-IR spectra of PEI and PBA-PEI. (**G**) Representative TEM image of PBA-PEI@siRNA nanocomposites. (**H**) Particle size of PBA-PEI@siRNA nanocomposites at different N/P ratios. (**I**) Zeta potential of PBA-PEI@siRNA nanocomposites at different N/P ratios. (**J**) Agarose gel electrophoresis (3%, *w*/*v*) of PBA-PEI@siRNA nanocomplexes. Lanes 1–6: naked siRNA, and PBA-PEI@siRNA at N/P ratios of 1, 2.5, 5, 7.5, and 10, respectively. *** *p* < 0.001; “ns” denotes no statistical significance (*p* > 0.05).

**Figure 2:**
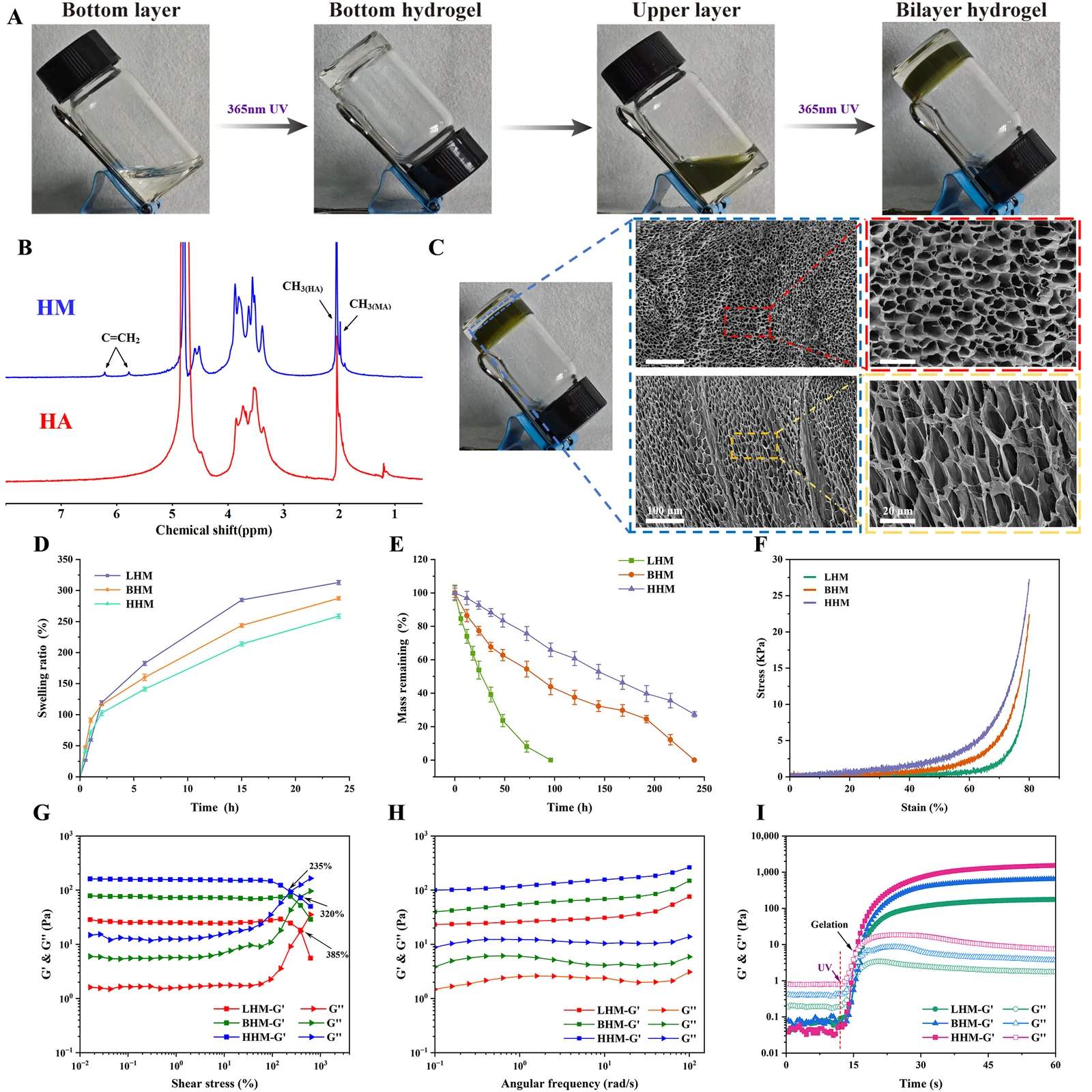
Preparation and physicochemical characterization of BHM@VP@PPs bilayer hydrogels. (**A**) Schematic illustration of the preparation process of BHM@VP@PPs bilayer hydrogels. (**B**) ^1^H NMR spectra of hyaluronic acid (HA) and methacrylate HA (HM). (**C**) Scanning electron microscopy (SEM) images of the BHM@VP@PPs bilayer hydrogels. (**D**) Swelling ratios of LHM, HHM, and BHM hydrogels. (**E**) Degradation profiles of LHM, HHM, and BHM hydrogels in PBS. (**F**) Compressive mechanical properties of LHM, HHM, and BHM hydrogels. (**G**) Variations in storage modulus ($G^{\prime }$) and loss modulus ($G^{\prime \prime }$) as a function of strain amplitude (0.01–1000%) at a constant angular frequency of 10 rad/s. (**H**) Variations in $G^{\prime }$ and $G^{\prime \prime }$ as a function of angular frequency (0.1–100 rad/s) at a constant strain of 1%. (**I**) Rheological properties of LHM, HHM, and BHM hydrogels under continuous UV irradiation.

**Figure 3:**
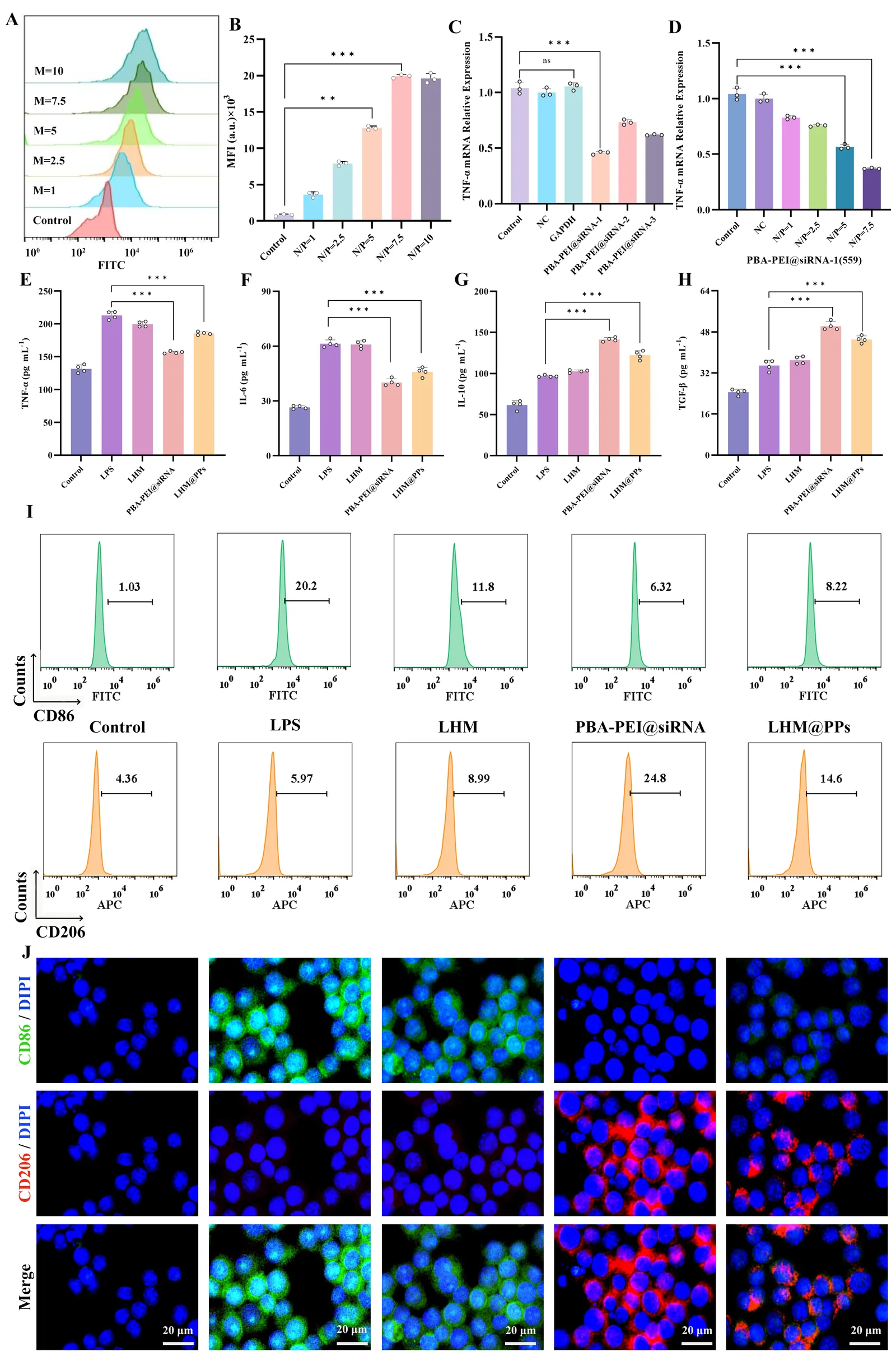
Evaluation of gene silencing efficiency and anti-inflammatory effects of PBA-PEI@siRNA nanocomposites and hydrogel-loaded nanocomposites on macrophage phenotype regulation. (**A**) Flow cytometry histograms showing the uptake of PBA-PEI@siRNA nanocomposites at different N/P ratios by RAW 264.7 cells. Green: FAM-labeled siRNA. (**B**) Quantitative analysis of the mean fluorescence intensity from the flow cytometry data shown in (**A**). (**C**) Gene silencing efficiency of different siRNA sequences targeting TNF-α. (**D**) Gene silencing efficiency of the selected sequence-1 (559) targeting TNF-α at different N/P ratios (*n* = 3). (**E**–**H**) Enzyme-linked immunosorbent assay (ELISA) of TNF-α protein levels in RAW 264.7 cells treated with sequence-1 (559) targeting TNF-α (*n* = 4). (**I**) Flow cytometry analysis of RAW 264.7 cells treated with sequence-1 (559) targeting TNF-α. (**J**) Immunofluorescence staining of RAW 264.7 cells treated with the sequence-1 (559) targeting TNF-α. ** *p* < 0.01, *** *p* < 0.001; “ns” denotes no statistical significance (*p* > 0.05).

**Figure 4:**
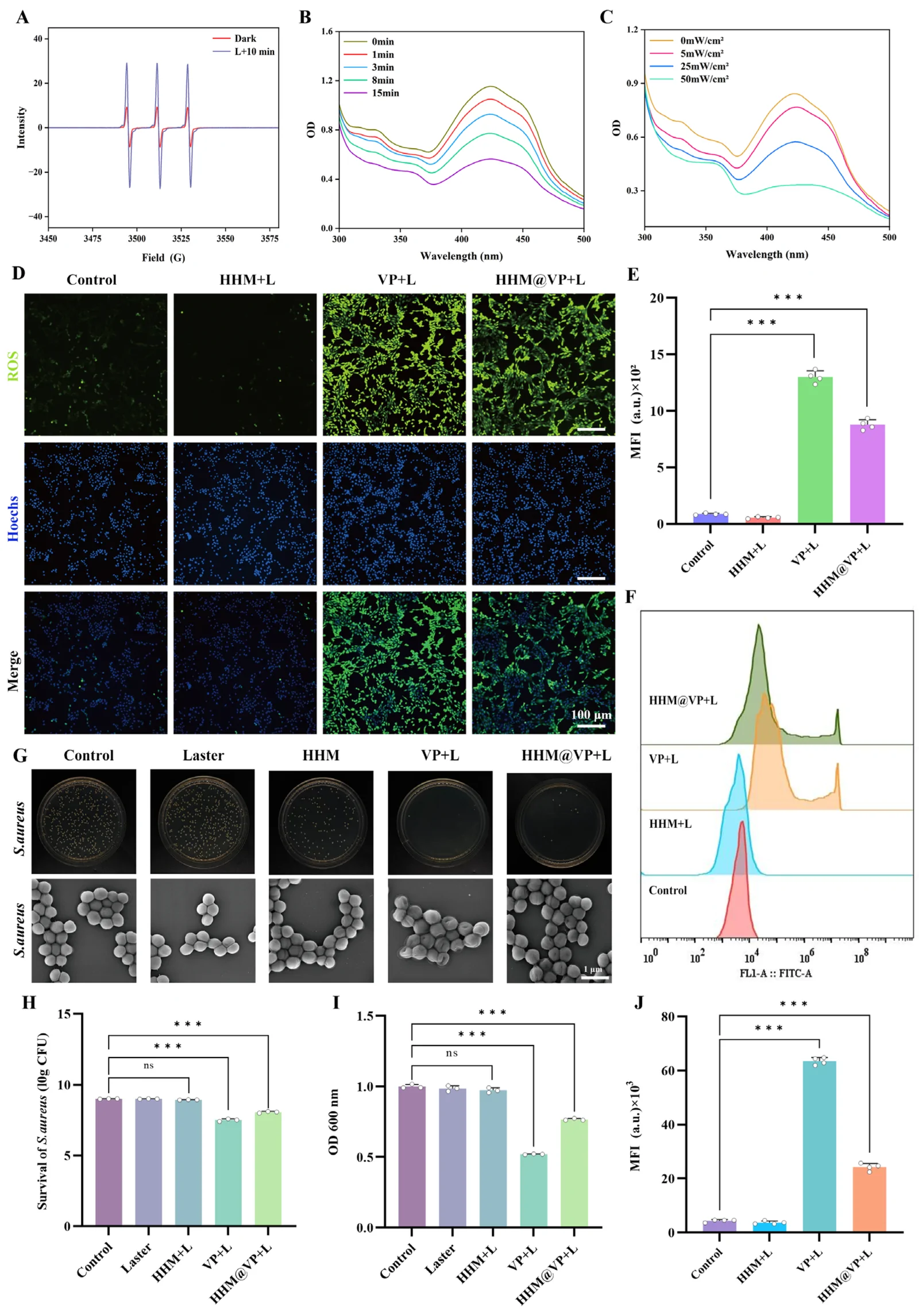
Characterization of verteporfin (VP) mediated photodynamic therapy (PDT) and evaluation of its antibacterial effects. (**A**) Generation of singlet oxygen (^1^O_2_) by VP before and after red light irradiation, as detected by electron paramagnetic resonance (EPR). (**B**) Singlet oxygen production by VP under different red light irradiation times using DPBF. (**C**) Singlet oxygen production by VP under different red-light intensities using DPBF. (**D**) Fluorescence images of intracellular reactive oxygen species (ROS) induced by VP-mediated PDT. (**E**) Quantitative analysis of the fluorescence intensity of reactive oxygen species (ROS) induced by VP-mediated PDT (*n* = 4). (**F**) Flow cytometric analysis of intracellular ROS induced by VP-mediated PDT. (**G**) Bacterial survival of *S. aureus* on agar plates after VP-mediated photodynamic therapy (PDT), and corresponding SEM morphological observations of bacterial colonies. (**H**) *S. aureus* survival rates following VP-mediated PDT (*n* = 3). (**I**) OD values of *S. aureus* suspensions after VP-mediated PDT at 660 nm (*n* = 3). (**J**) Quantitative analysis of ROS fluorescence intensity induced by VP-mediated PDT using flow cytometry (*n* = 4). *** *p* < 0.001; “ns” denotes no statistical significance (*p* > 0.05).

**Figure 5:**
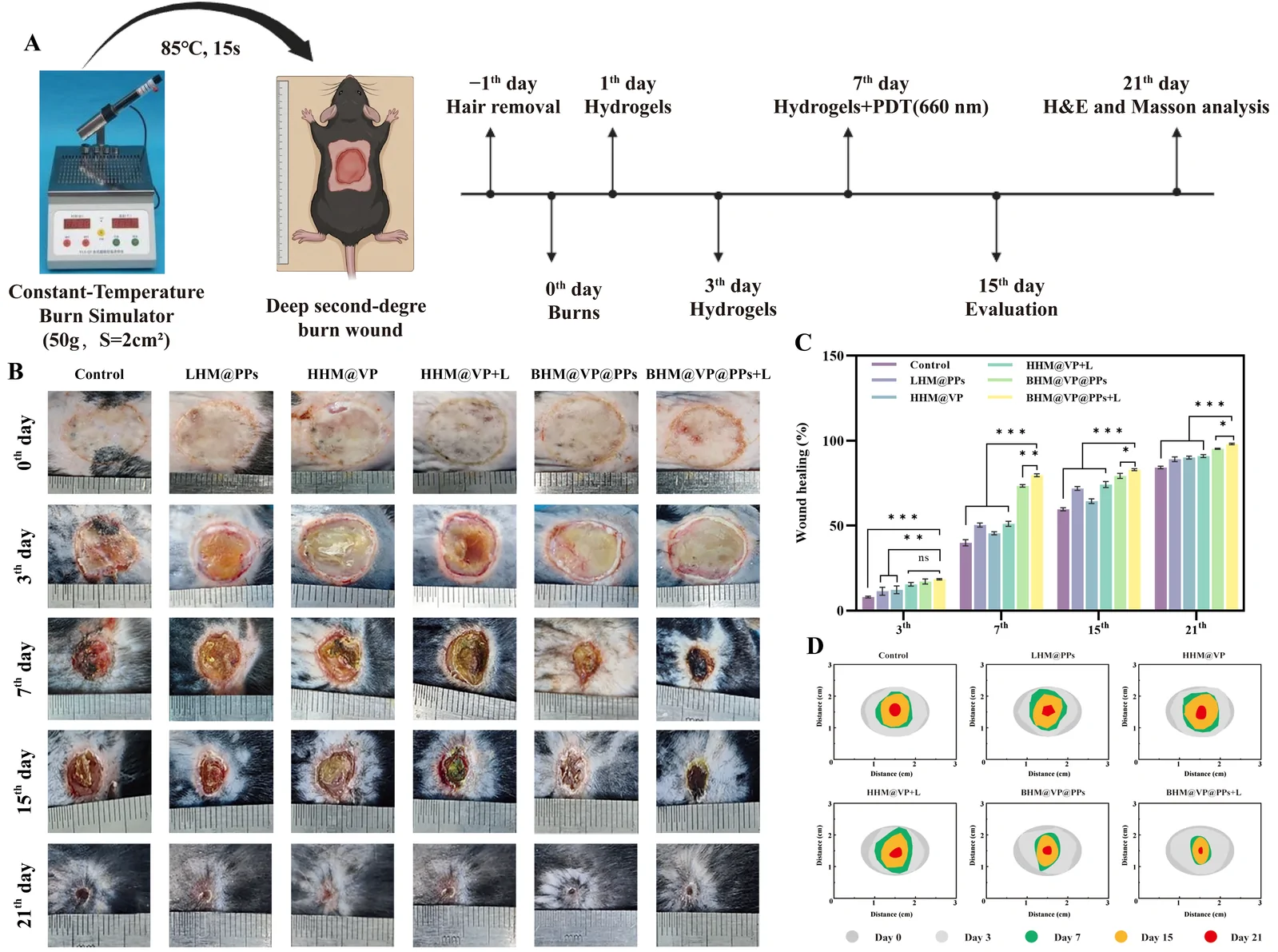
Promotion of scar-minimizing healing of deep second-degree burn wounds by the BHM@VP@PPs hydrogel. (**A**) Schematic diagram illustrating the establishment of the deep second-degree burn wound model, treatment protocol, and tissue collection timeline. (**B**) Representative photographs of dorsal skin wounds in mice at indicated time points after different treatments. Scale bar: 2 mm. (**C**) Wound healing rates calculated on days 3, 7, 15, and 21 post-treatment. (**D**) Quantitative analysis of the relative wound area on the mouse dorsum at different time points. * *p* < 0.05, ** *p* < 0.01, *** *p* < 0.001. (*n* = 5); “ns” denotes no statistical significance (*p* > 0.05).

**Figure 6:**
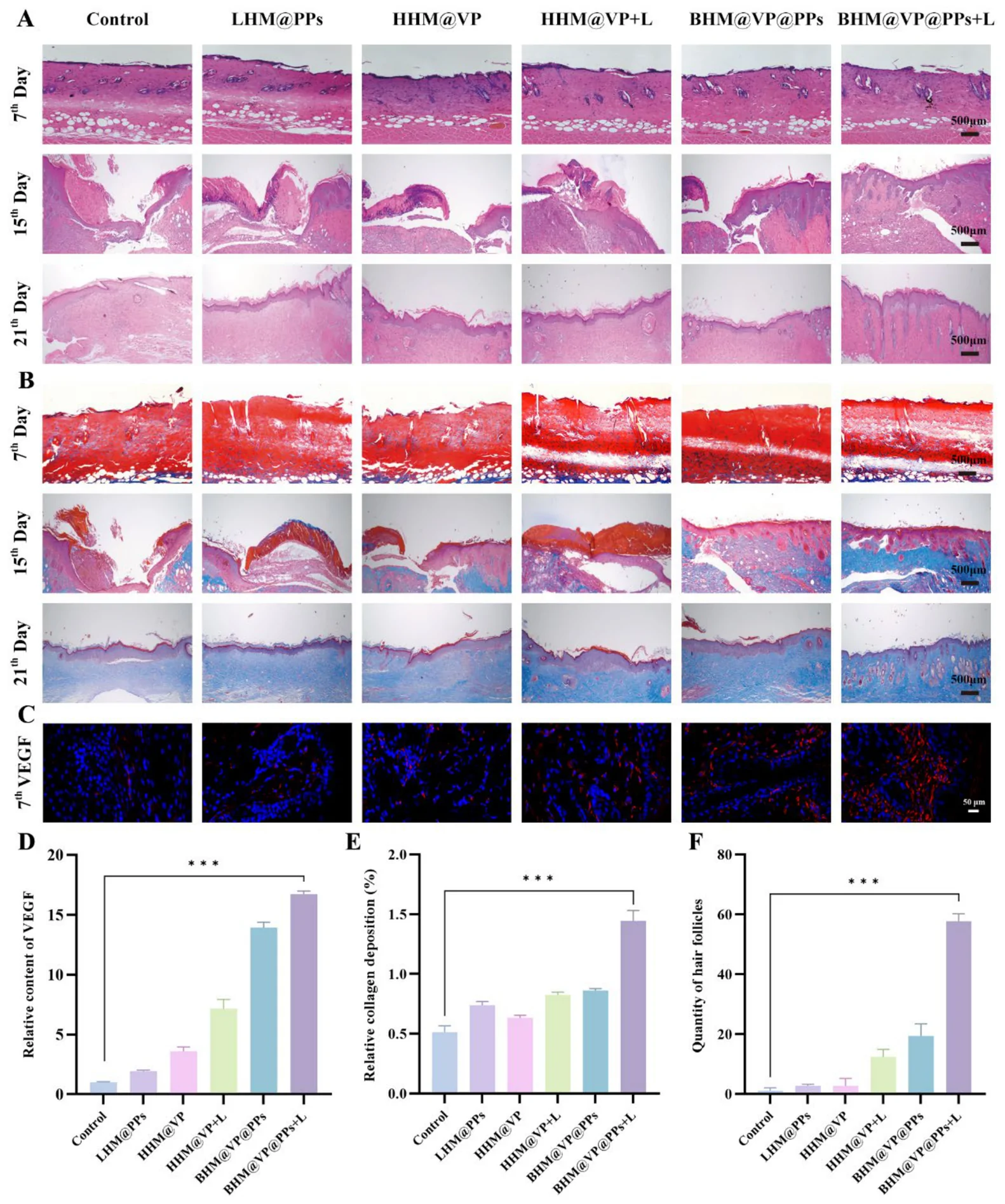
Histological evaluation of wound tissue. (**A**) Hematoxylin and eosin (H&E) and (**B**) Masson’s trichrome staining of regenerated wounds in the six treatment groups on days 7, 15, and 21 post-treatment. (**C**) Immunofluorescence assessment of vascular endothelial growth factor (VEGF) in the six groups on day 7. (**D**) Quantitative analysis of VEGF levels, (**E**) collagen deposition, and (**F**) hair follicle count in burn wound tissue on day 21. *** *p* < 0.001. (*n* = 5).

**Figure 7:**
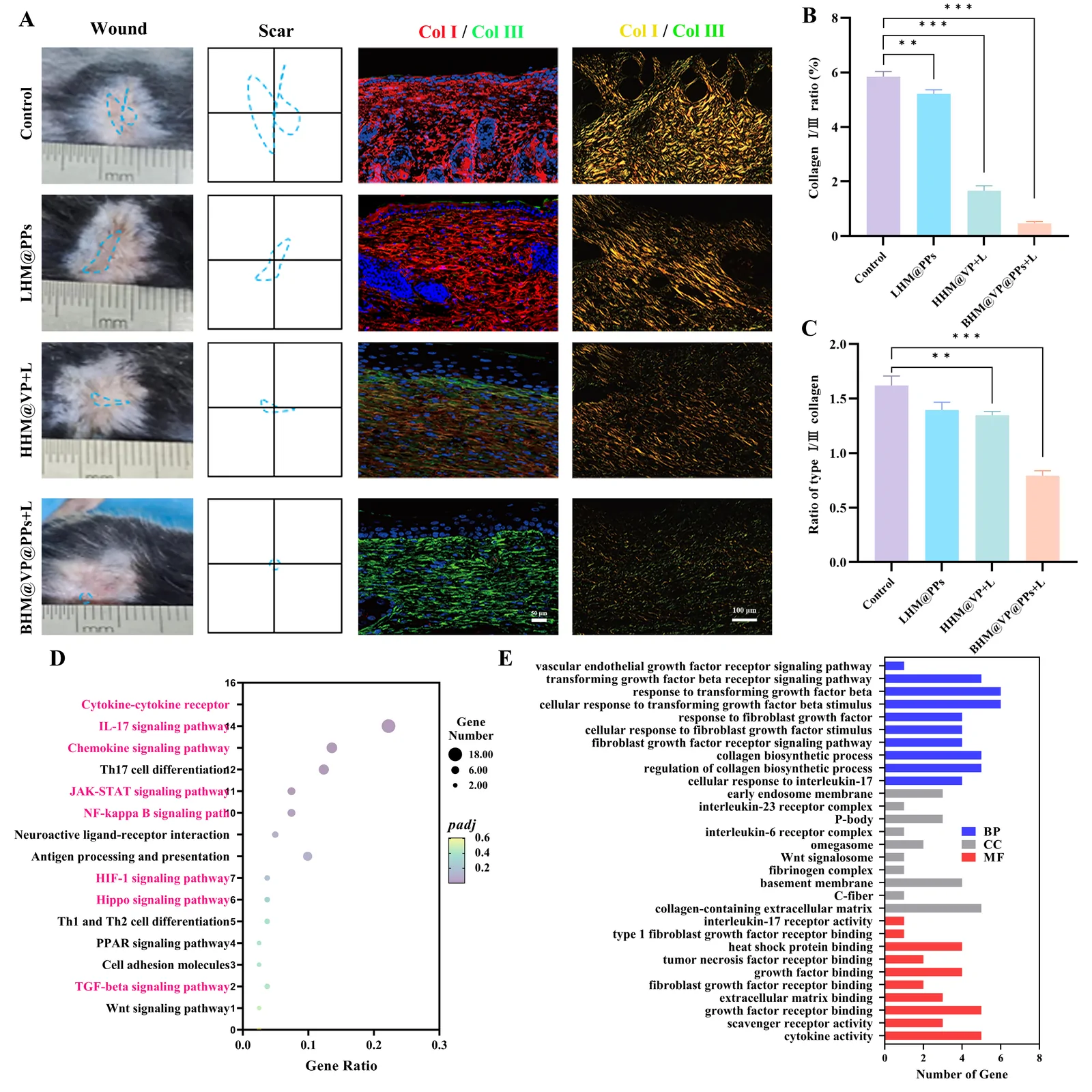
Evaluation of the anti-scarring effect of the BHM@VP@PPs hydrogel on deep second-degree burn wounds on the mouse dorsum. (**A**) Representative photographs of wounds on day 28, scar simulation images, immunofluorescence staining of collagen type I (COL I) and collagen type III (COL III) and polarized light microscopy images after Sirius Red staining. (**B**) COL I/COL III ratio determined by immunofluorescence staining. (**C**) COL I/COL III ratio determined by polarized light microscopy after Sirius Red staining. (**D**) KEGG pathway enrichment analysis and (**E**) GO functional enrichment analysis of differentially expressed genes. ** *p* < 0.01, *** *p* < 0.001 (*n* = 5).

**Scheme 1:**
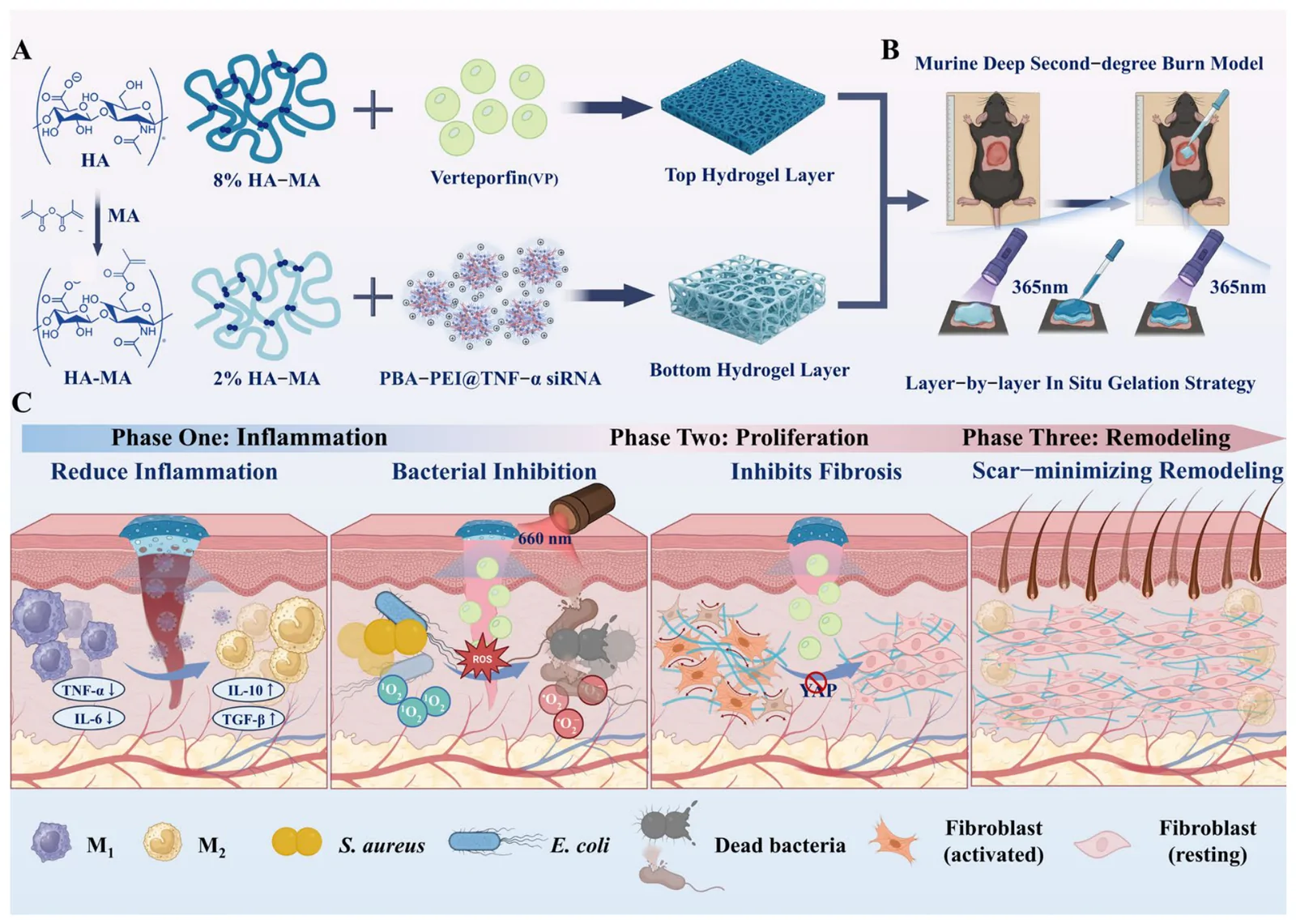
Schematic illustration of the synthesis of the BHM@VP@PPs bilayer hydrogel and its proposed mechanism for scar-minimizing healing of deep second-degree burn wounds on the mouse dorsum. (**A**) Flowchart of the bilayer hydrogel preparation process. (**B**) Establishment of a deep second-degree burn model on the mouse dorsum and the corresponding treatment protocol. (**C**) Proposed mechanism of action of the BHM@VP@PPs bilayer hydrogel in mediating scar-minimizing healing of deep second-degree burn wounds on the mouse dorsum.

## Data Availability

The original contributions presented in this study are included in the article/Supplementary Materials. Further inquiries can be directed to the corresponding authors.

## Supplementary Materials

The following supporting information can be downloaded at: https://www.mdpi.com/article/10.3390/gels12100887/s1, Table S1: Results of polymer element distribution. Table S2: siRNA sequences targeting the TNF-α gene. Table S3: Primer sequences for RT-qPCR analysis of relevant genes. Figure S1: Evaluation of the serum stability of PBA-PEI@siRNA nanocomposites. Figure S2: Quantitative analysis of serum stability. Figure S3: Fourier-transform infrared spectroscopy (FT-IR) spectrum of HM. Figure S4: Rheological properties of hydrogels. Figure S5: Fluorescence image of PBA-PEI@siRNA with different N/P ratio-transfected RAW 264.7 cells. Figure S6: Transfection efficiency of PBA-PEI@siRNA with different N/P ratios. Figure S7: Release profile of siRNA and Verteporfin. Figure S8: ROS generation of VP generated under 660 nm laser irradiation (25 mW/cm^2^) in vitro. Figure S9: Full wavelength spectrum of VP before and after irradiation with 660 nm laser for 30 min. Figure S10: Generation of superoxide anions by VP before and after red light irradiation detected by electron paramagnetic resonance. Figure S11: Full wavelength profile of VP under different red-light exposure times (25 mW/cm^2^). Figure S12: Full wavelength profile of VP under different red-light intensities (15 min). Figure S13: Evaluation of the antibacterial efficacy of VP-mediated PDT. Figure S14: Cell viability after treatment with various concentrations of verteporfin. Figure S15: Western blot analysis of YAP and α-SMA protein levels in fibroblasts treated with different concentrations of VP (0, 1, 2, 5 μg/mL). Figure S16: Quantitative analysis of YAP and α-SMA protein expression from Western blotting in fibroblasts treated with different concentrations of VP (*n* = 3). Figure S17: Biocompatibility of hydrogels in vivo. H&E staining images of major organs (heart, liver, spleen, lung and kidney) in mice on day 28 (*n* = 5). Figure S18: Volcano plot of transcriptomic analysis of differentially expressed genes (*n* = 5).

## Abbreviations

The following abbreviations are used in this manuscript

HA	Hyaluronic acid
HM	Methacrylate hyaluronic acid
siRNA	Small interfering RNA
DPBF	1,3-Diphenylisobenzofuran
DCFH-DA	2′,7′-Dichlorodihydrofluorescein diacetate
DEPC	Diethypyrocarbonate
ROS	Reactive oxygen species
OD	Optical density
G′	storage modulus
G″	loss modulus
EPR	Electron paramagnetic resonance
PDT	Photodynamic therapy
FT-IR	Fourier transform infrared
^1^H NMR	^1^H Nuclear magnetic resonance
*S. aureus*	*Staphylococcus aureus*
*E. coli*	*Escherichia coli*
H&E	Hematoxylin and Eosin
Masson	Masson’s Trichrome Staining
