# Supplementary material for: Biomimetic Bilayer Hydrogel for Scar-Minimizing Healing of Deep Second–Degree Burns Through Sequential Macrophage–Fibroblast Intervention
Source: Gels. 2026 Oct 1;12(10):887. doi: 10.3390/gels12100887 (PMC13704041; doi:10.3390/gels12100887)
Supplement: Supplementary file 1 [file gels-12-00887-s001.zip › gels-4555585-supplementary.pdf]

Supporting Information:

Biomimetic Bilayer Hydrogel for Scar-Minimizing Healing of Deep Second−Degree Burns Through Sequential Macrophage−Fibroblast Intervention

Jian Jia [1,2,3], Jiale Wang [1,2,3], Wenjing Yan [1,2,3], Maosen Yang [1,2,3], Zixuan Zhang [1,2,3], Chaoting Gao [1,2,3], Yang Li [1,2,3,*] and Daidi Fan [1,2,3,*]

1 Engineering Research Center of Western Resource Innovation Medicine Green Manufacturing, Ministry of Education, School of Chemical Engineering, Northwest University, Xi'an 710127, China

2 Shaanxi Key Laboratory of Biomaterials and Synthetic Biology, Shaanxi R&D Center of Biomaterials and Fermentation Engineering, School of Chemical Engineering, Northwest University, Xi'an 710127, China

3 Biotech. & Biomed. Research Institute, Northwest University, Xi'an 710127, China

* Correspondence: liyang2023@nwu.edu.cn (Y.L.); fandaidi@nwu.edu.cn (D.F.)

Table S1. Results of polymer element distribution.

Sample	C (%)	H (%)	N (%)
PEI	55.8	11.6	32.5
PBA−PEI	47.15	8.94	21.55

Table S2. siRNA sequences targeting the TNF−α gene.

Name	Sequence (5'−3')
559−TNF−α sense	GAUGGGUUGUACCUUGUCUTT
559−TNF−α antisense	AGACAAGGUACAACCCAUCTT
644−TNF−α sense	GAUUUGCUAUCUCAUACCATT
644−TNF−α antisense	UGGUAUGAGAUAGCAAAUCTT
729−TNF−α sense	GAGCUCAAACCCUGGUAUGTT
729−TNF−α antisense	CAUACCAGGGUUUGAGCUCTT
GAPDH sense	CACUCAAGAUUGUCAGCAATT
GAPDH antisense	UUGCUGACAAUCUUGAGUGAG
NC sense	UUCUCCGAACGUGUCACGUTT
NC antisense	ACGUGACACGUUCGGAGAATT

Table S3. Primer sequences for RT−qPCR analysis of relevant genes.

Gene	Primer	Sequence (5'−3')
TNF−α	Forward	AGTGGAGGAGCAGCTGGAGT
	Reverse	TCCCAGCATCTTGTGTTTCTG
GAPDH	Forward	CATGCCGCCTGGAAACCTGCCA
	Reverse	TGGGCTGGGTGGTCCAGGGGTTTC

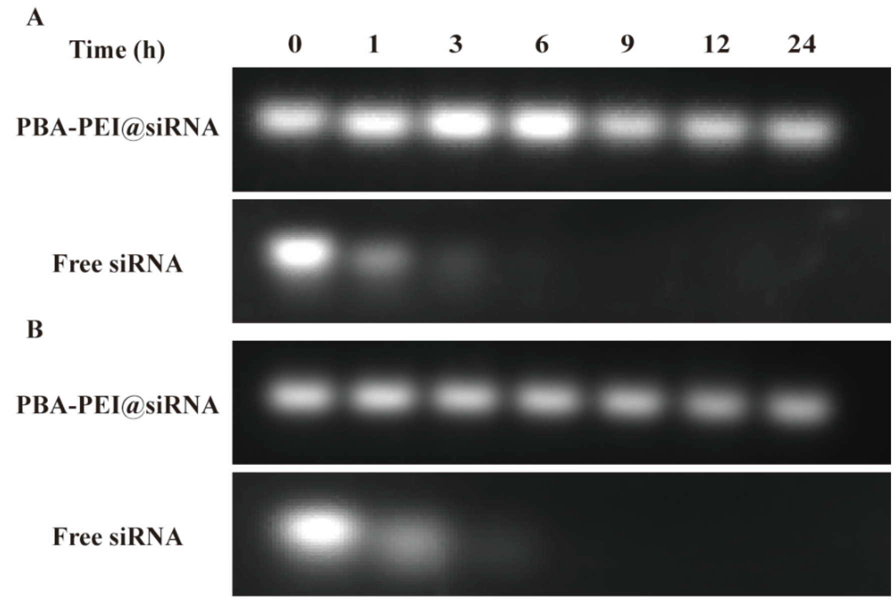

Figure S1. Evaluation of the serum stability of PBA−PEI@siRNA nanocomposites. (A) Stability in 10% fetal bovine serum; (B) Stability in 50% fetal bovine serum.

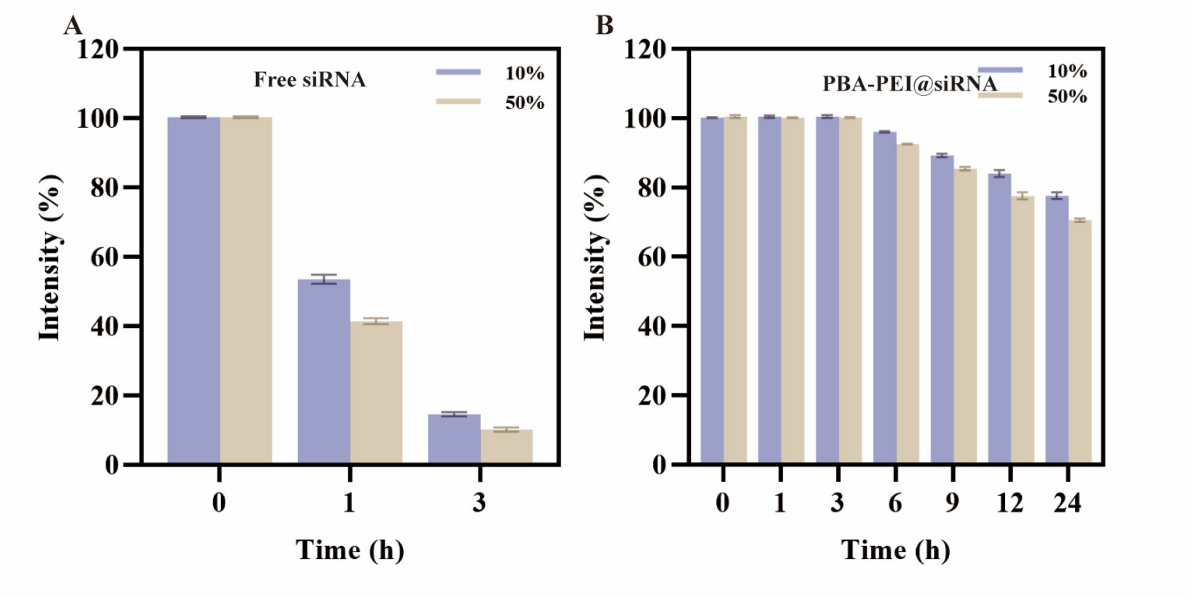

Figure S2. Quantitative analysis of serum stability. (A) Free siRNA; (B) PBA−PEI@siRNA nanocomposites.

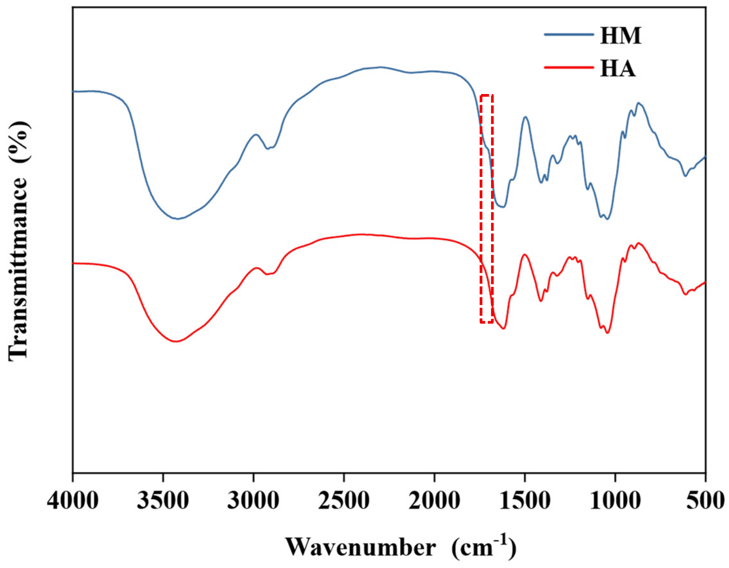

Figure S3. Fourier−transform infrared spectroscopy (FT−IR) spectrum of HM.

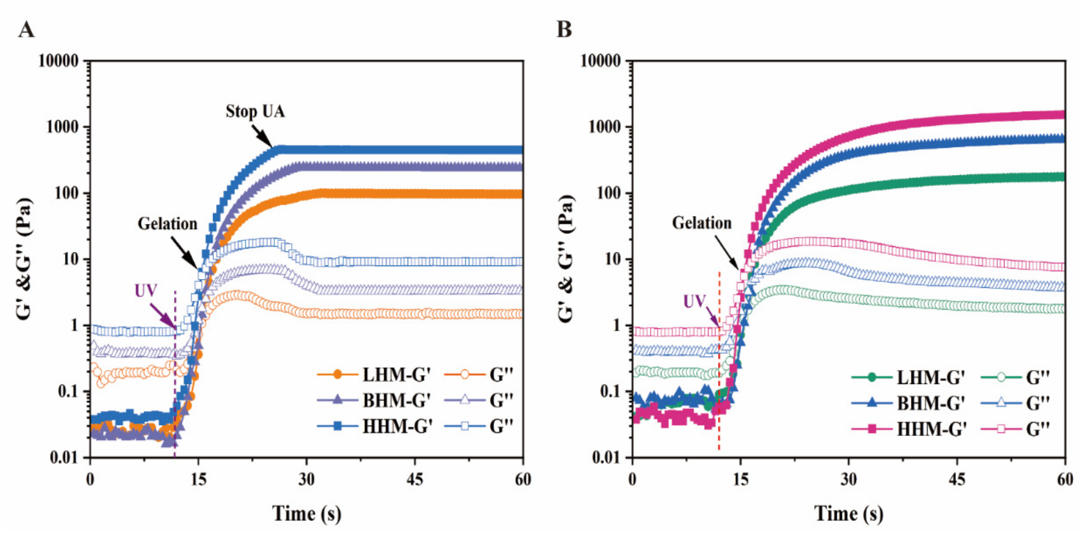

Figure S4. Rheological properties of hydrogels: (A) Under continuous UV irradiation; (B) After 12 seconds of UV irradiation.

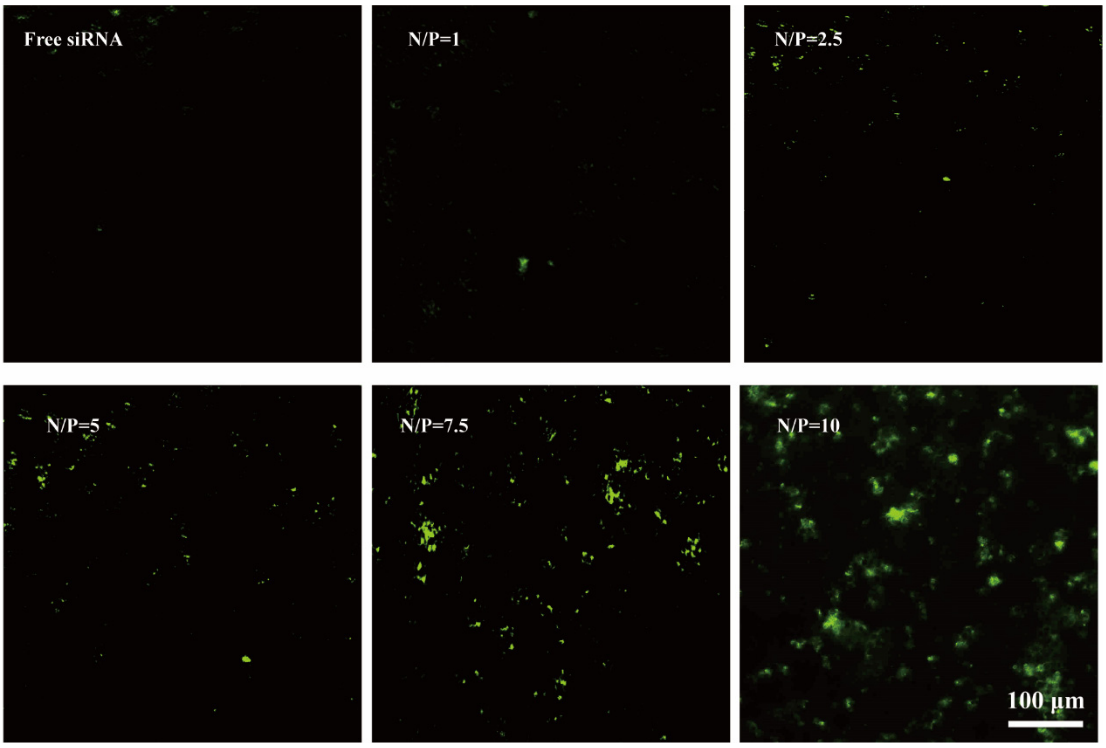

Figure S5. Fluorescence image of PBA−PEI@siRNA with different N/P ratio–transfected RAW 264.7 cells. Green: FAM−labeled siRNA.

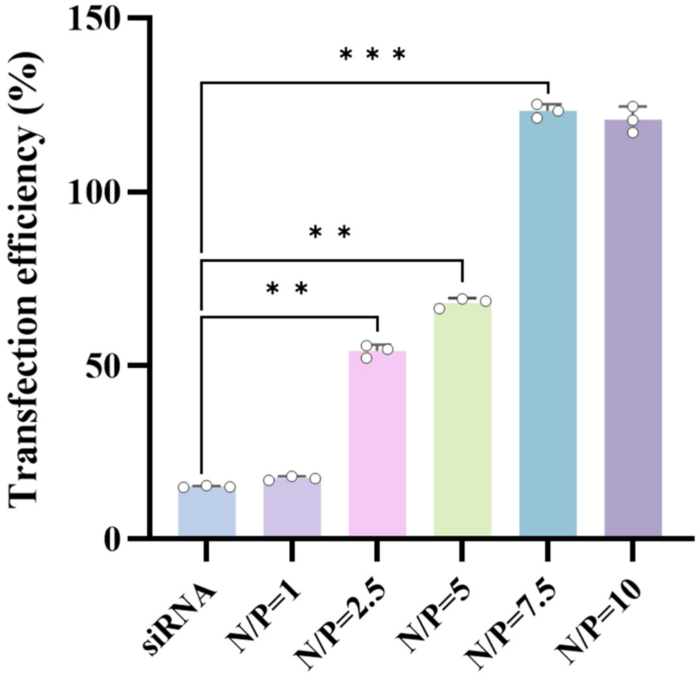

Figure S6. Transfection efficiency of PBA−PEI@siRNA with different N/P ratios. $^{**}p < 0.01$, $^{***}p < 0.001$.

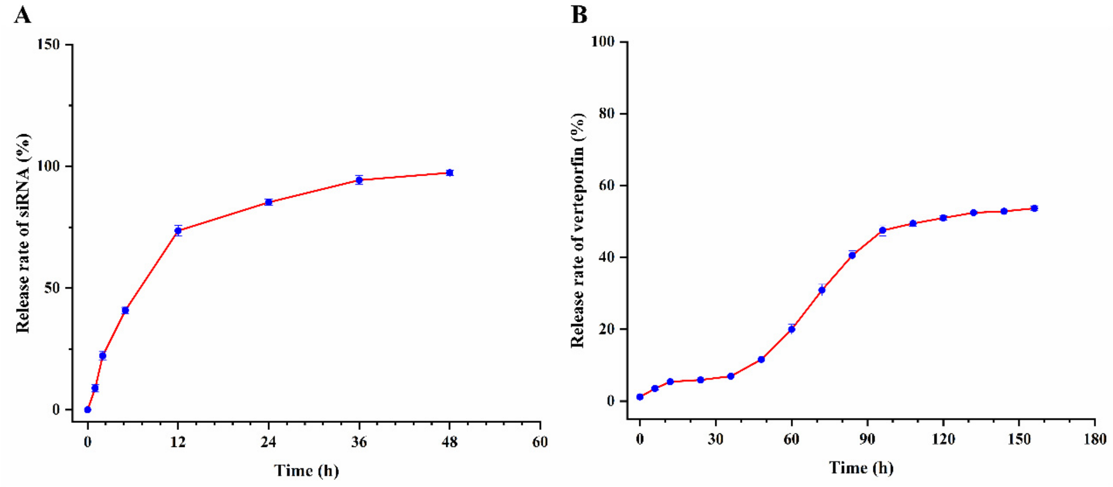

Figure S7. Release profile of siRNA (A) and Verteporfin (B).

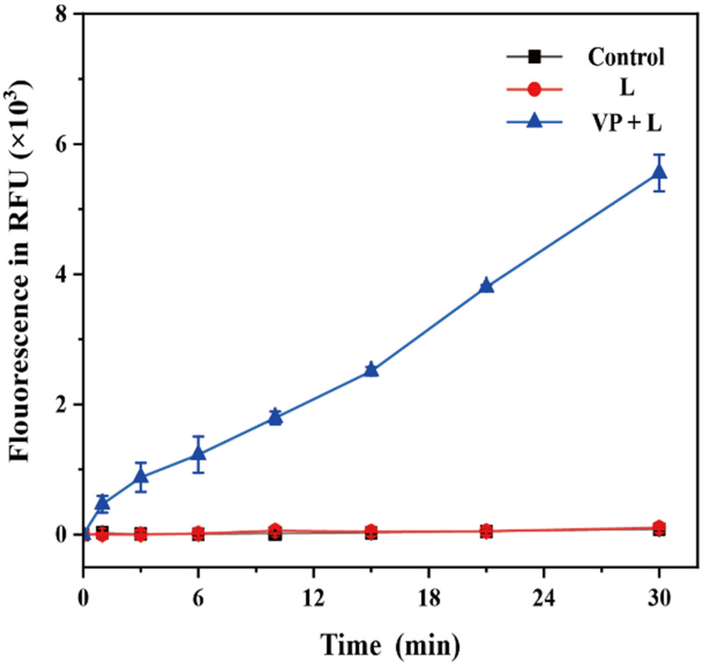

Figure S8. ROS generation of VP generated under 660 nm laser irradiation (25mW/cm^2) in vitro.

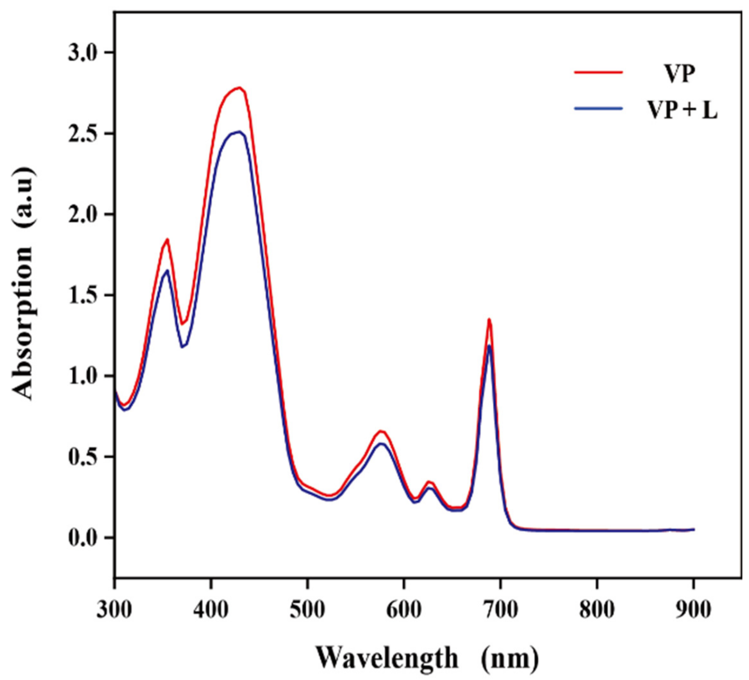

Figure S9. Full wavelength spectrum of VP before and after irradiation with 660 nm laser for 30 min.

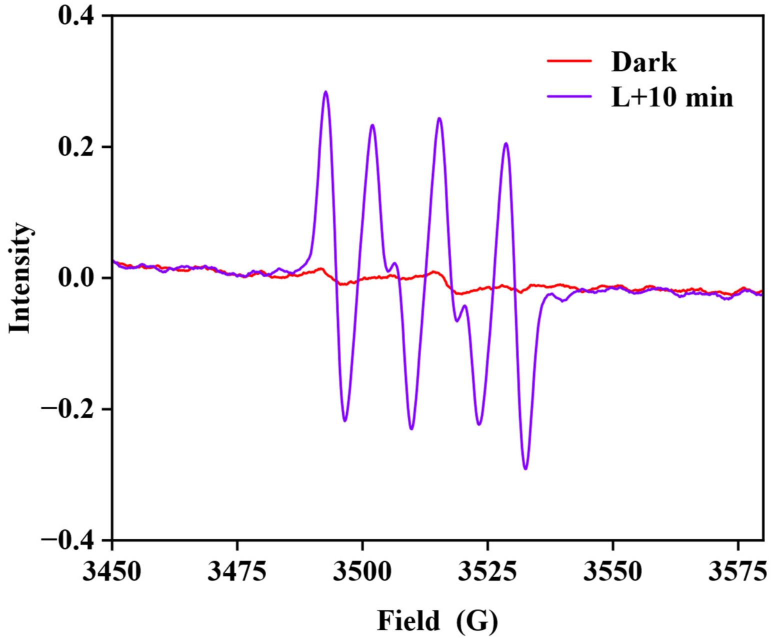

Figure S10. Generation of superoxide anions by VP before and after red light irradiation detected by electron paramagnetic resonance.

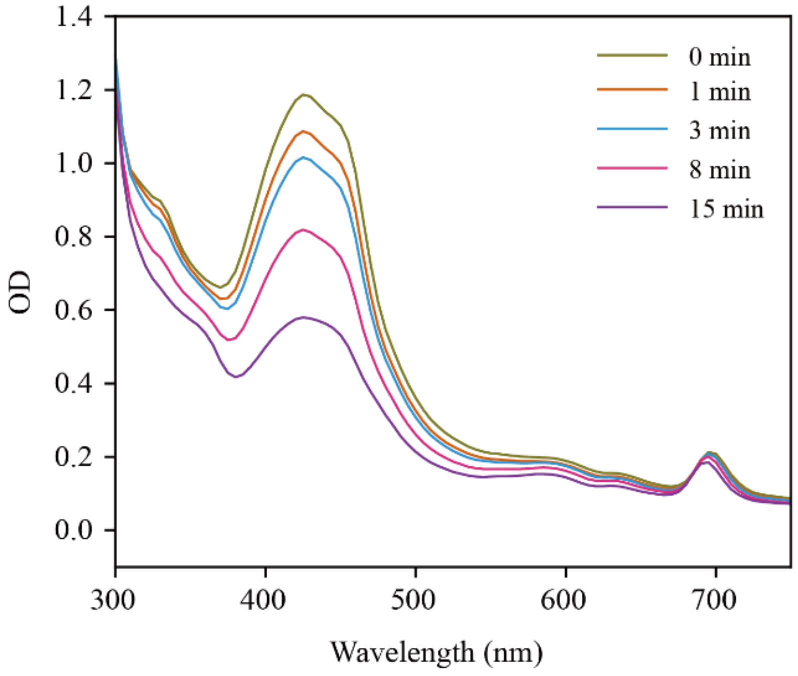

Figure S11. Full wavelength profile of VP under different red−light exposure times (25mW/cm²).

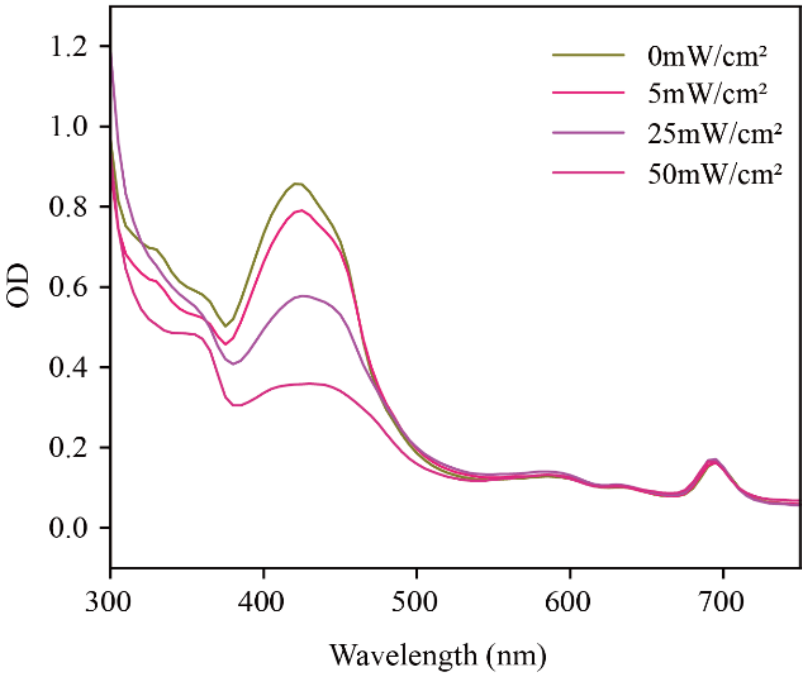

Figure S12. Full wavelength profile of VP under different red−light intensities (15min).

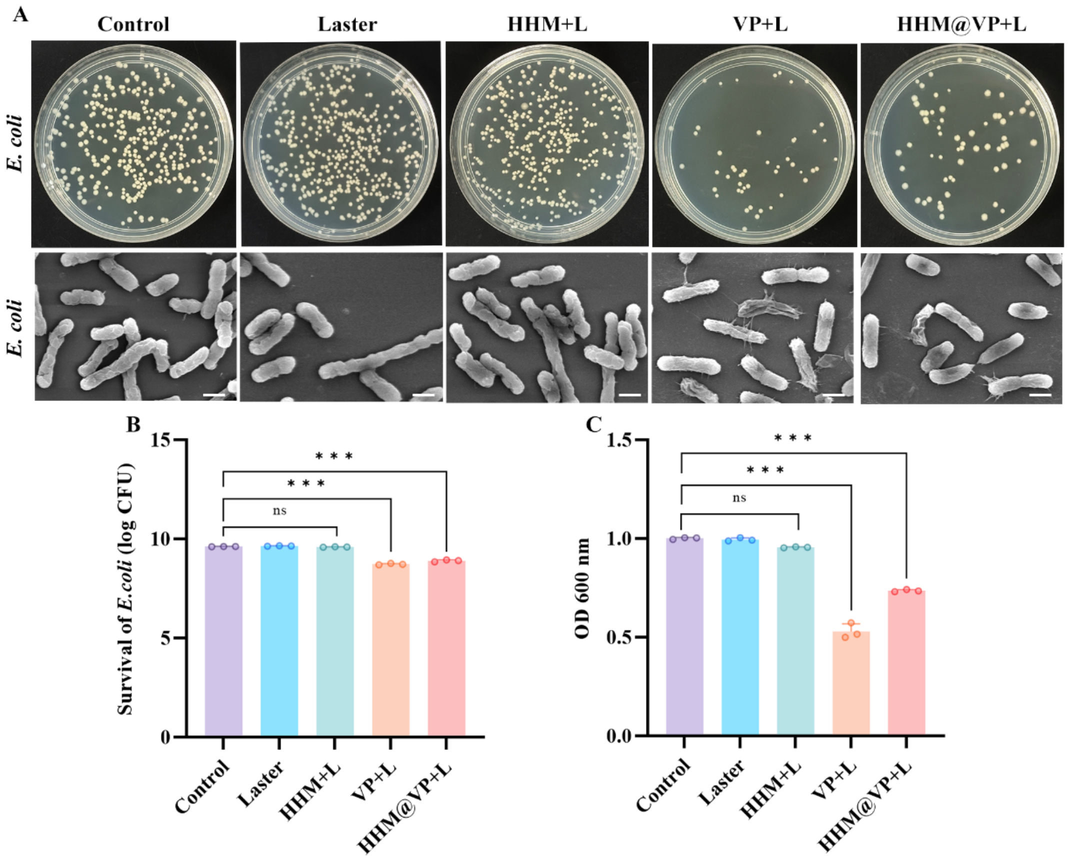

Figure S13. (A) Evaluation of the antibacterial efficacy of VP-mediated PDT. (B) *E. coli* survival rates following VP-mediated PDT ($n = 3$). (C) OD values of *E. coli* s suspensions after VP−mediated PDT at 660 nm ($n = 3$). ***$p < 0.001$, Scale bar: 0.5μm.

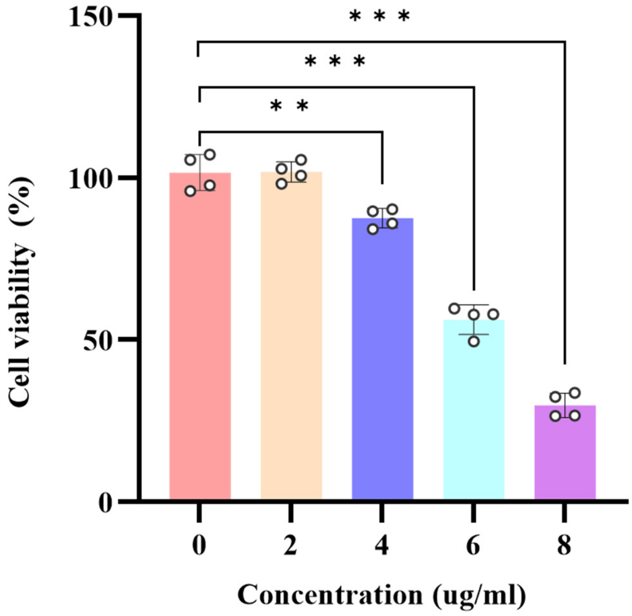

Figure S14. Cell viability after treatment with various concentrations of verteporfin (n = 4). **p< 0.01, ***p < 0.001.

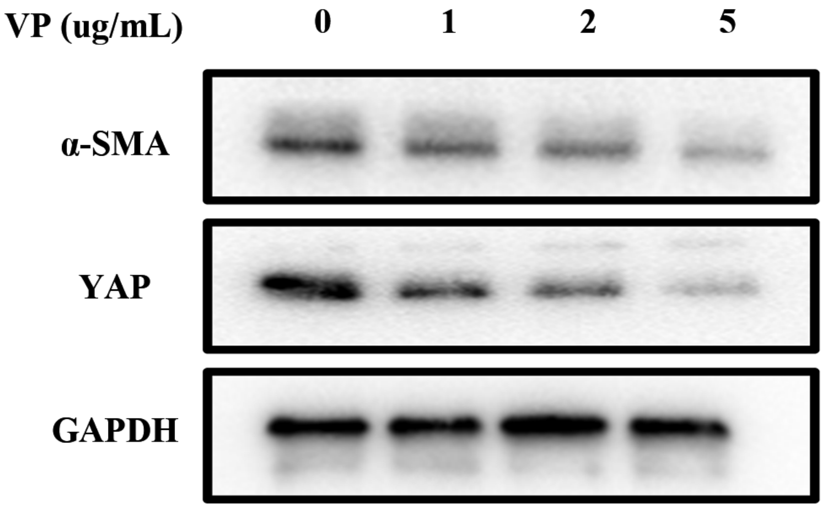

Figure S15. Western blot analysis of YAP and α−SMA protein levels in fibroblasts treated with different concentrations of VP (0, 1, 2, 5 μg/mL).

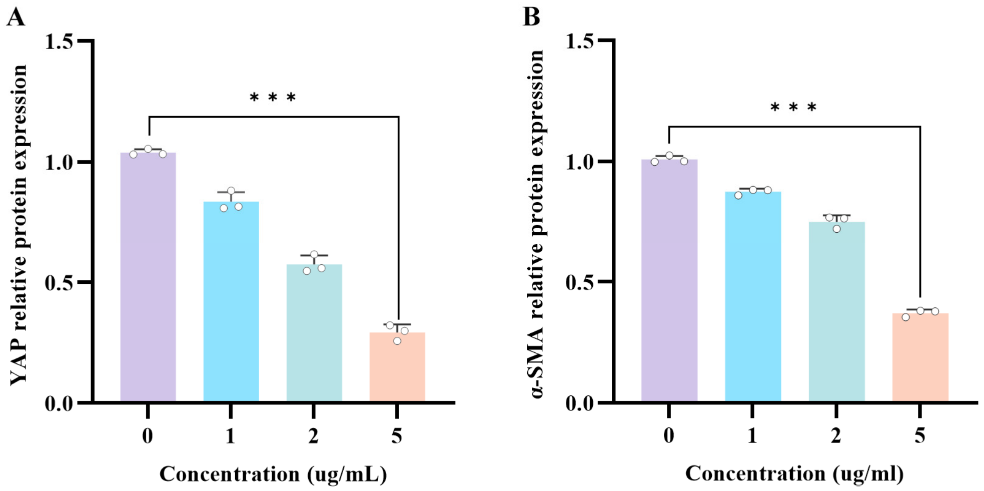

Figure S16. Quantitative analysis of YAP (A) and α−SMA (B) protein expression from Western blotting in fibroblasts treated with different concentrations of VP (n = 3). ***p < 0.001.

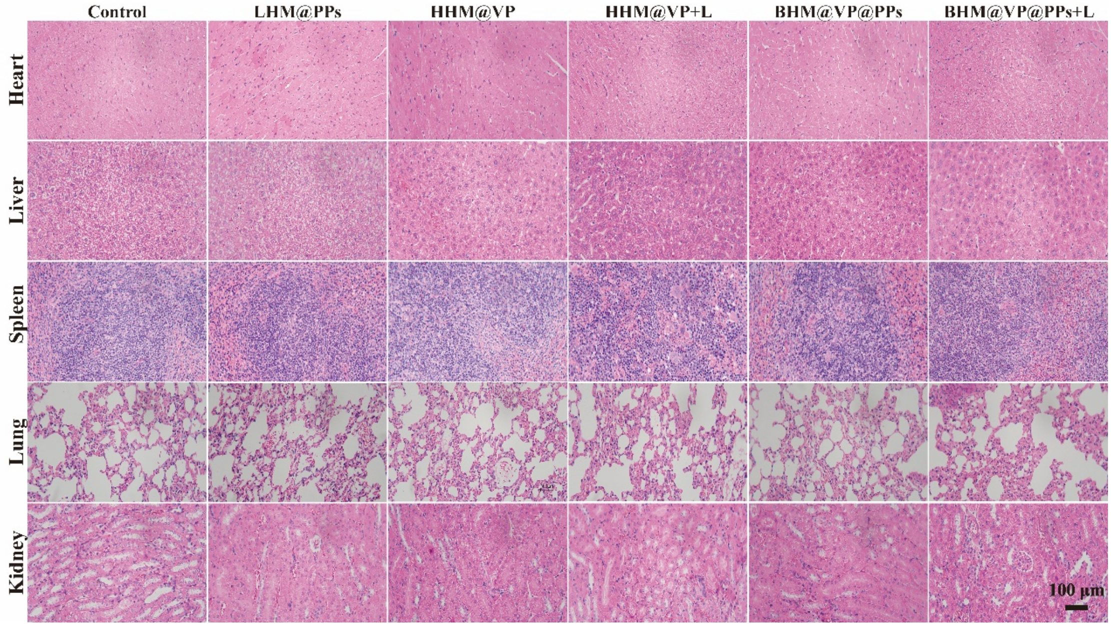

Figure S17. Biocompatibility of hydrogels in vivo. H&E staining images of major organs (heart, liver, spleen, lung and kidney) in mice on day 28 (n = 5).

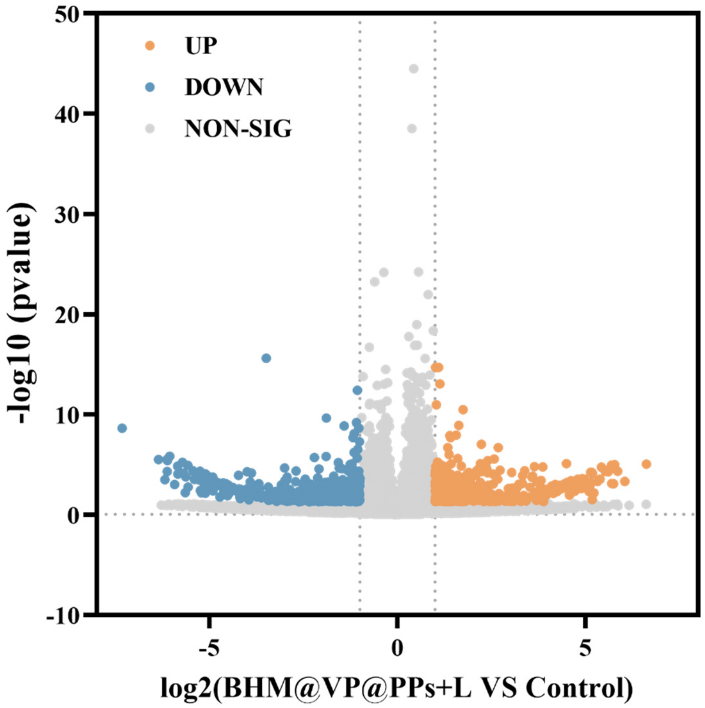

Figure S18. Volcano plot of transcriptomic analysis of differentially expressed genes (*n* = 5).
